# Swimming Upstream to Understand Congenital Anomalies of the Kidney and Urinary Tract: Zebrafish Models for Developmental Biology, Disease Mechanisms, and Functional Interpretation of Genetic Variation

**DOI:** 10.3390/genes17080867

**Published:** 2026-07-24

**Authors:** Zachary W. Nurcombe, Lina Mougharbel, Thomas M. Kitzler

**Affiliations:** 1 Department of Human Genetics, McGill University, 845 Sherbrooke St. W, Montreal, QC H3A 0G4, Canada; zachary.nurcombe@mail.mcgill.ca; 2 Research Institute of the McGill University Health Centre, 1001 Blvd. Décarie, Montreal, QC H4A 0B1, Canada; lina.mougharbel@affiliate.mcgill.ca; 3 Department of Specialized Medicine, Division of Medical Genetics, McGill University Health Centre, 1001 Blvd. Décarie, Montreal, QC H4A 0B1, Canada

**Keywords:** CAKUT, zebrafish, congenital anomalies of the kidney and urinary tract, renal development, pronephros, monogenic disease, disease modelling, variant interpretation, functional genomics, kidney morphogenesis

## Abstract

Congenital anomalies of the kidney and urinary tract (CAKUT) are the leading cause of pediatric chronic kidney disease (CKD) and comprise a heterogeneous group of developmental disorders with a substantial genetic contribution. Advances in next-generation sequencing have facilitated the identification of numerous candidate genes and rare variants associated with CAKUT. However, establishing causality and defining the biological functions of implicated genes remain major challenges. Functional validation is therefore essential to bridge the gap between gene discovery and mechanistic understanding, enabling the interpretation of genetic variation within the context of kidney development and disease. The zebrafish (*Danio rerio*) has emerged as a powerful in vivo model for studying renal development and interrogating the function of CAKUT-associated genes. Its utility stems from a high degree of genetic and developmental conservation with humans, conserved nephrogenic pathways, optical transparency during embryogenesis, and the relative ease of genetic manipulation. In this review, we provide an overview of zebrafish kidney development within the broader context of vertebrate nephrogenesis, highlighting the key genetic programs governing intermediate mesoderm specification, nephron segmentation, and pronephric morphogenesis. We then systematically examine CAKUT-associated genes that have been modeled in zebrafish, focusing on studies that have linked genetic perturbations to renal development and structural phenotypes. Finally, we discuss the strengths and limitations of zebrafish models for functional genomics and variant interpretation and consider their emerging role in bridging genetic discovery with mechanistic insights into CAKUT pathogenesis.

## 1. Introduction

**Figure 1:**
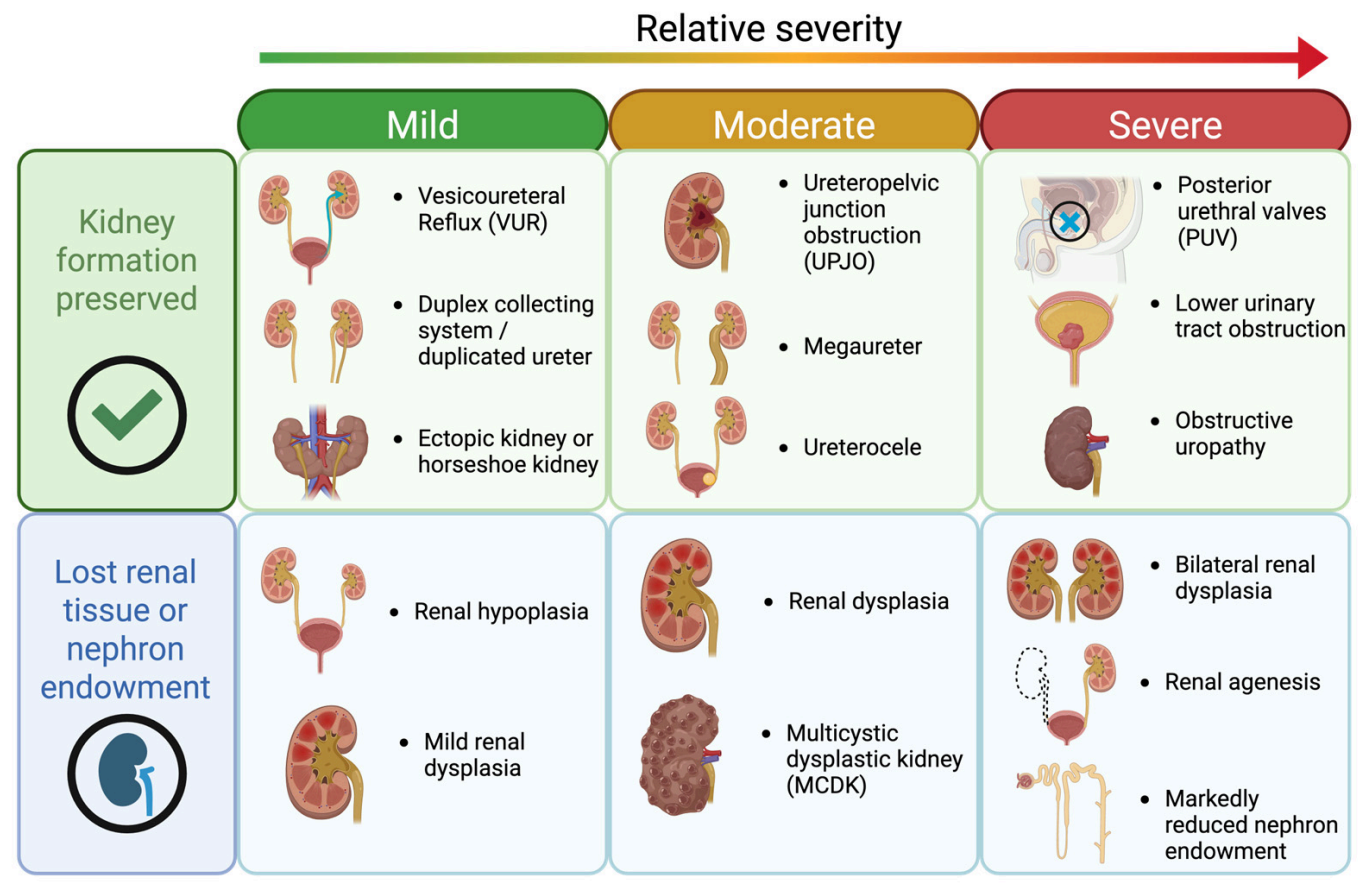
Severity spectrum of congenital anomalies of the kidney and urinary tract (CAKUT) phenotypes. Representative phenotypes organized by severity and whether kidney formation is preserved or lost. This highlights the broad heterogeneity of CAKUT phenotypes. Created in BioRender. Nurcombe, Z. (2026) https://BioRender.com/g0q0qtp.

Congenital anomalies of the kidney and urinary tract (CAKUT) are the most common cause of chronic kidney disease (CKD) in children and account for a substantial portion of pediatric kidney failure [1]. Although individual renal and urinary tract malformations have long been recognized as a distinct clinical phenomenon, the acronym CAKUT first appeared in the literature in the late 1990s as a term to describe congenital defects affecting the kidney and urinary tract [2,3]. This terminology signaled a shift away from viewing these phenotypes as separate entities, and instead toward a developmental framework in which diverse renal and urinary tract anomalies could arise from the disruption of shared processes during embryogenesis [3,4]. CAKUT arises during embryogenesis as a result of perturbations in the signaling networks that coordinate kidney and urinary tract organogenesis, including reciprocal interactions between the ureteric bud and the metanephric mesenchyme [5,6]. Depending on timing, location, and nature of these perturbations, CAKUT can manifest as a broad spectrum of phenotypes. At the severe end of the spectrum, developmental defects may result in a marked reduction in renal tissue or nephron endowment, leading to phenotypes such as renal agenesis, renal hypodysplasia, and multicystic dysplastic kidney. In other cases, kidney formation is largely preserved, but structural anomalies may predispose affected individuals to chronic renal injury. These manifestations include ureteropelvic and ureterovesical junction obstructions, megaureter, vesicoureteral reflux, posterior urethral valves, or collecting system duplication anomalies [6] (Figure 1). The remarkable phenotypic heterogeneity of CAKUT is mirrored by its complex genetic architecture, in which diverse genetic and environmental factors converge on shared developmental pathways governing nephrogenesis and urinary tract morphogenesis.

CAKUT can be caused by maternal and environmental factors, but approximately 20% of cases can be attributed to an identifiable monogenic or chromosomal cause [7,8,9]. A conservative estimate suggests that at least 54 genes have been implicated as monogenic causes of CAKUT, resulting in both syndromic and non-syndromic phenotypes [5]. Many of these genes encode transcription factors, signaling molecules, extracellular matrix (ECM) components, and other proteins required for kidney and urinary tract development. Therefore, the genetic architecture of CAKUT reflects the coordinated developmental programs that govern renal specification, morphogenesis, and urinary tract formation.

Next-generation sequencing has facilitated the identification of numerous candidate CAKUT genes and rare variants; however, establishing genotype–phenotype relationships and determining the pathogenicity of these variants remain major challenges [6,10,11]. In many cases, the precise role of a candidate gene in kidney development remains elusive, including the stage of organogenesis at which it functions, the cell populations in which it is expressed, and how its disruption affects developmental programs and tissue morphogenesis [10]. These challenges are compounded by the observation that many CAKUT-associated genes exhibit autosomal dominant inheritance with reduced penetrance and variable expressivity, such that pathogenic variants may be inherited from apparently unaffected individuals [6,12]. This phenomenon has been well documented for established CAKUT genes such as *HNF1B* and *PAX2*, in which both intrafamilial and interfamilial phenotypic variability can be observed, even among individuals carrying the same pathogenic variant [13,14,15,16]. Consequently, segregation analyses frequently require large, carefully phenotyped pedigrees and may still be insufficient to establish causality.

Despite the increasing adoption of next-generation sequencing technologies, the genetic basis of many CAKUT cases remains unresolved, with fewer than 30% of affected individuals receiving a molecular diagnosis [17,18]. Although whole-exome sequencing (WES) has improved diagnostic yield compared with targeted gene panels, most studies identify only a limited number of pathogenic variants, suggesting that additional disease genes, genetic mechanisms, and regulatory pathways remain to be discovered [17,19]. Hence, functional evidence is required to distinguish causal variants from rare background genetic variation, determine candidate gene involvement in CAKUT, and define how genetic perturbations disrupt renal development and establish candidate-gene involvement and variant pathogenicity [10,20,21].

This need for functional validation is particularly important because CAKUT arises from the perturbation of complex early developmental processes that depend on precisely coordinated signaling between multiple progenitor populations, including the ureteric bud and metanephric mesenchyme [5,8]. Timing, dose, and location can result in the spectrum of phenotypes seen in patients with a CAKUT phenotype, and penetrance and variable expressivity can hinder conclusions regarding causation [6,8,22]. Robust experimental systems are therefore needed to test candidate genes and variants of uncertain significance (VUS) in vivo and to investigate spatiotemporal developmental processes, including nephron patterning, cell signaling, tissue morphogenesis, and urinary tract development. An ideal model system should enable rapid functional interrogation of candidate genes and variants, facilitate the study of early renal development in vivo, and provide reproducible phenotypic readouts that can be linked to developmental disease mechanisms.

Studying CAKUT genes in vivo requires a model that enables the investigation of early kidney development, tissue-tissue signaling, and conserved cellular pathways that are essential for renal development [5,22,23]. Although mammalian models such as mice and rats are invaluable for studying CAKUT genes, they are not well suited for high-throughput functional testing of novel candidate genes and variants because of their relatively small litter sizes, long generation times, and high maintenance costs [24]. By contrast, zebrafish provide a powerful complementary model with several advantages. First, zebrafish embryos develop quickly ex utero, allowing direct observation of embryogenesis and organ development [24,25]. The pronephros is established by approximately 24 hours post fertilization (hpf), with glomerular filtration commencing by 48 hpf [26,27]. The transparency of zebrafish embryos enables direct visualization of the pronephros throughout development and facilitates the use of labeling techniques such as immunohistochemistry and in situ hybridization. In addition, a single mating produces hundreds of embryos, allowing large experimental sample sizes and high-throughput studies [24]. Finally, zebrafish are readily amenable to genetic manipulation, making them a powerful model for investigating the function of candidate CAKUT genes and assessing the effects of genetic variants [21,24,27]. Although zebrafish kidney development has been studied for more than two decades, studies investigating CAKUT-associated genes using zebrafish models remain relatively limited and are scattered throughout the literature. This review synthesizes the available evidence, provides a framework for interpreting zebrafish phenotypes in the context of human CAKUT phenotypes, and identifies priorities for future functional studies.

## 2. Zebrafish Kidney Development and Conservation

**Figure 2:**
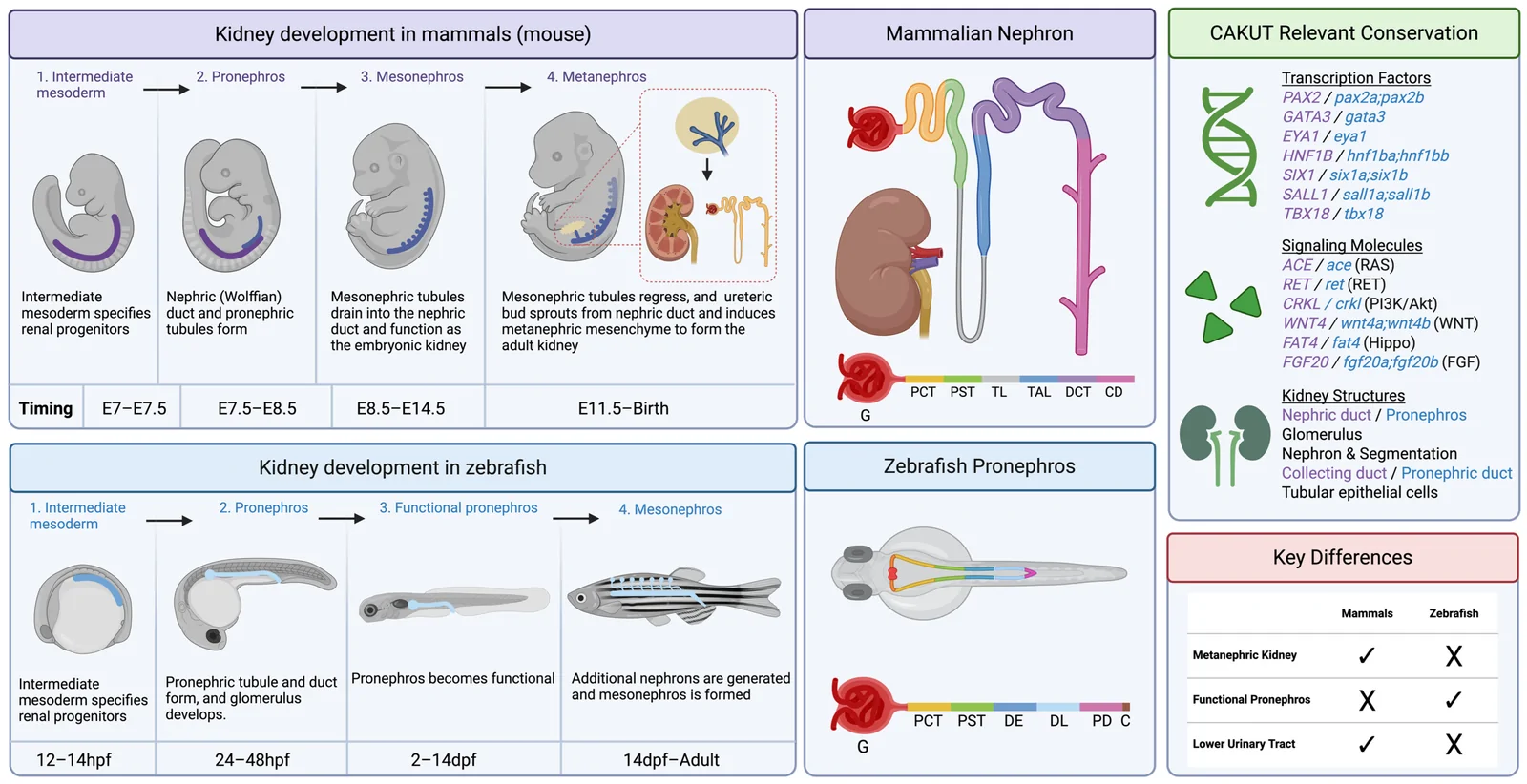
Mammalian vs. zebrafish renal development. Comparison of mammalian and zebrafish renal development highlights the differences and similarities between mammals and zebrafish. Unlike mammals, zebrafish do not form a metanephric kidney, but they share many conserved structures, signaling molecules, and architecture C: cloaca, CD: collecting duct, DCT: distal convoluted tubule, DE: distal early tubule, DL: distal late tubule, dpf: days post fertilization, E: embryonic, G: glomerulus, hpf: hours post fertilization, PCT: proximal convoluted tubule, PD: pronephric duct, PST: proximal straight tubule, TAL: thick ascending limb of Henle, TL: thin limb of Henle. The mammalian and zebrafish nephron segmentation schematics were adapted from Elmonem et al. (2018) [42]. Created in BioRender. Nurcombe, Z. (2026) https://BioRender.com/x7yqskz.

Evolution has conserved many of the developmental programs that govern vertebrate kidney formation. Renal development begins with the specification of the intermediate mesoderm (IM), the formation of the nephric duct, followed by nephrogenesis and nephron segmentation [28,29]. In mammals, these processes occur through three successive developmental stages: the pronephros, mesonephros, and metanephros, the latter giving rise to the permanent adult kidney [28,30] (Figure 2). In teleost fish, embryonic kidney development produces a functional pronephros, which is later supplemented and replaced by a mesonephric kidney during adulthood [27,31,32] (Figure 2). Despite these anatomical differences, many of the molecular pathways that regulate renal specification, nephric duct development, nephron patterning, and epithelial morphogenesis are highly conserved between zebrafish and mammals [30,32,33]. Because numerous CAKUT-associated genes function within these early developmental programs, zebrafish provide a powerful system for investigating conserved mechanisms of kidney development and for assessing the developmental consequences of genetic variation implicated in human CAKUT.

### 2.1. Intermediate Mesoderm

Specification of the IM is a highly conserved process that is essential for kidney development, as it gives rise to renal progenitor cells [28,30]. Numerous factors contribute to IM specification and patterning, among which *PAX2* and *PAX8* are key regulators that initiate renal lineage specification and nephrogenesis [28,34]. Zebrafish possess orthologs of these genes, *pax2a* and *pax8*, which perform analogous functions during kidney development. Loss of either of these genes results in defects in IM specification and subsequent kidney development, highlighting the conservation of these regulatory pathways across vertebrates [35,36]. In addition to *PAX2* and *PAX8*, IM specification is regulated by signaling pathways such as retinoic acid and bone morphogenetic protein (BMP) signaling, both of which have conserved roles in mammals and zebrafish [28,32,36,37,38]. Other conserved regulators of renal progenitor identity include LHX1, OSR1, and WT1 [39,40,41]. Disruption of these factors can impair IM specification and patterning.

### 2.2. The Nephric Duct

The next major step in renal development is formation of the nephric duct, a paired epithelial structure that extends caudally from the IM [29,43]. The nephric duct is a developmental structure shared between mammals and fish. In zebrafish, the pronephric duct serves as the embryonic drainage duct [44]. In mammals, this duct gives rise to the ureteric bud, which undergoes reciprocal signaling interaction with the metanephric mesenchyme, an intermediate mesoderm-derived tissue, to initiate formation of the metanephric kidney [29,45]. This process is tightly regulated by several genes such as *GATA3*, *RET*, *HNF1B*, *FGF*, and *WNT* [43,45,46,47]. Zebrafish have an ortholog for each of these mammalian genes, but importantly, zebrafish do not undergo ureteric bud morphogenesis and ureteric bud induction. Consequently, these processes cannot be modeled directly in zebrafish. Nevertheless, many genes involved in ureteric bud induction and branching retain a conserved function in zebrafish pronephric duct morphogenesis, allowing investigators to study the upstream developmental pathways that regulate nephric duct formation and epithelial morphogenesis, even in the absence of a metanephric kidney [33,36,46,48].

### 2.3. Nephron Segmentation

Nephron segmentation is the final conserved process during development of the renal system [30,32,33]. In the mammalian kidney, the tissue differentiates into the glomerulus (podocytes), proximal tubule, loop of Henle, distal tubule, and the collecting duct [30,49]. In contrast, the zebrafish nephron has distinct proximal and distal tubules, as well as a biologically collecting duct-like segment [32,33]. These segments are identified by the expression of shared markers. This includes, but is not limited to, *wt1a/b* labeling the glomerulus, *lrp2a* labeling the proximal tubule, *slc12a1* labeling the distal tubule, and *gata3* labeling the collecting duct/cloaca [32,43,50,51]. Zebrafish nephrons do not contain a loop of Henle [33]. Despite the simplicity of the zebrafish pronephros and mesonephros compared to a mammalian metanephros, the zebrafish nephron shares the segmentation and epithelial specialization seen in the mammalian nephron [32,33,42]. These segments provide experimentally tractable readouts when studying nephron development in zebrafish, and phenotypes observed can highlight patterning or specification problems.

### 2.4. Fundamental Differences Between Mammals and Zebrafish Kidneys

There are fundamental differences between the development and structure of the zebrafish renal system compared to mammals. First, mammals develop a metanephros through ureteric budding, which induces branching by signaling to the metanephric mesenchyme [29,30]. Zebrafish instead develop an embryonic pronephros consisting of two bilateral nephrons by 24 hpf, with function established by 48 hpf, and develop a mesonephric kidney as adults rather than a metanephric kidney [27,31,33,48]. Therefore, zebrafish cannot be used to model processes and mechanisms specific to the metanephros such as ureteric bud induction/branching or renal pelvis and ureter development. Despite these differences, the core developmental molecules and pathways are conserved between both systems such as nephron patterning, epithelial specification, and glomerular filtration formation [27,32,33]. Zebrafish are relevant for studying conserved processes such as progenitor differentiation, nephron segmentation, epithelial polarity, tubule morphogenesis, duct opening, ECM and basement membrane (BM) integrity, as well as filtration. For these reasons, the zebrafish pronephros provides an excellent model for studying conserved developmental processes relevant to CAKUT [27,33].

## 3. CAKUT Genes Modeled in Zebrafish

Several genes implicated in human CAKUT have been investigated using the zebrafish model (Table 1). While the depth of functional characterization varies considerably across studies, ranging from gene knockdown to expression studies, they provide useful insights into understanding the mechanistic role of these genes in renal development. In this section, we summarize CAKUT genes that have been examined in zebrafish with a particular focus on renal expression, kidney-specific phenotypes, and the relevance to human phenotype and disease.

Among the established CAKUT-associated genes, a subset has been investigated in zebrafish through approaches ranging from gene expression studies to functional analyses involving gene knockdown, knockout, or other forms of genetic perturbation followed by assessment of renal developmental phenotypes [5]. In this section, we focus on genes for which zebrafish studies have provided evidence of renal expression and/or functional involvement in kidney development. A comprehensive list of human CAKUT-associated genes curated by Kolvenbach et al. (2023), including genes for which little or no zebrafish data are currently available, is provided in Table S1 [5].

### 3.1. Transcription Factors

Many genes implicated in CAKUT encode transcription factors, which represent the most extensively studied class of CAKUT-associated genes in zebrafish. Renal phenotypes resulting from the disruption of transcription factors in zebrafish most commonly manifest as defects in pronephric morphogenesis, nephron specification, and nephron segmentation [36,46]. These phenotypes are particularly informative because many transcription factors function within developmental pathways that are highly conserved between zebrafish and mammals. Consequently, alterations in nephron patterning and renal morphogenesis provide robust developmental readouts for assessing the roles of CAKUT-associated genes and identifying mechanisms that may contribute to human disease.

Examples of transcription factors studied in zebrafish are *PAX2*, *HNF1B*, and *GATA3*. *PAX2* is a well-established CAKUT-associated gene in which pathogenic variants can cause syndromic renal hypoplasia and a spectrum of other renal and extrarenal manifestations (MIM# 120330). In zebrafish, the ancestral *PAX2* gene underwent duplication, resulting in the orthologs *pax2a* and *pax2b*. Both genes play important roles during early kidney development, and disruption of *pax2a* in particular leads to defects in IM specification, nephric duct development, and nephron formation. Groups focused on *pax2a* as the primary zebrafish ortholog and found that it is expressed early in the zebrafish [36,38]. Loss of *pax2a* function in zebrafish, as observed in the *no isthmus* mutant, results in defective pronephric differentiation accompanied by abnormalities of the distal pronephric duct and cloaca [36]. These findings highlight the conserved role of *PAX2* in nephric duct development and nephron patterning across vertebrates. *HNF1B*, which has also been duplicated in zebrafish (*hnf1ba/hnf1bb*), was thoroughly investigated with embryonic expression of both orthologs [38,46,65]. *hnf1ba* zebrafish knockouts develop pronephric cysts and pericardial edema, with disrupted tubule development [66]. Simultaneous knockout and knockdown of *hnf1ba* and *hnf1bb* results in broadly disrupted tubular differentiation, and segmentation [46]. In the developing zebrafish pronephros, *gata3* is expressed in the distal pronephric duct [32,38]. Studies investigating *gata3* function in zebrafish have found that loss of *gata3* results in abnormalities of the distal pronephric duct, accompanied by loss of *ret* expression in homozygous mutants [67]. These findings support a conserved role for *GATA3* in nephric duct development and suggest that disruption of the *GATA3–RET* developmental axis may contribute to CAKUT pathogenesis.

Several additional CAKUT-associated transcription factors have also been investigated in zebrafish. For example, perturbation of *bnc2* and *six2a/six2b* results in renal-specific phenotypes such as glomerular cysts, outlet obstructions, and tubule morphogenesis defects [57,68]. Collectively, these studies demonstrated that disruption of transcription factors involved in kidney development most commonly manifests as abnormalities in renal progenitor specification, nephron segmentation, tubule differentiation, and epithelial morphogenesis. The consistency of these phenotypic outcomes reflects the conserved roles of transcription factors in coordinating nephrogenic programs across vertebrate species. Although these studies have established useful developmental readouts for assessing CAKUT-associated transcription factors in zebrafish, several genes implicated in human renal disease have not yet been associated with a characterized renal phenotype in this model. Examples include but are not limited to *hoxa11a/hoxa11b*, *foxp1a/foxp1b, six5,* and *sox17* (Full list can be found in Table S1). The absence of zebrafish renal phenotyping data for these genes highlights an important gap in the field and suggests that additional functional studies may further expand our understanding of the developmental pathways underlying CAKUT.

### 3.2. Developmental Signaling and Pronephric Morphogenesis Molecules

Many CAKUT-associated genes are implicated in renal developmental signaling pathways that regulate pronephric morphogenesis, segmentation, and epithelial organization. As kidney and urinary tract development relies on coordinated signals between epithelial and mesenchymal tissues, these genes are of particular relevance. Typically, these genes were studied in the context of nephron patterning and duct morphogenesis in zebrafish rather than ureteric bud branching. Notably, distal pronephric duct phenotypes are a recurring feature of many CAKUT zebrafish models, underscoring their relevance to human renal development. Although the zebrafish distal pronephric duct is not anatomically equivalent to the mammalian collecting system, it shares developmental origins and conserved signaling pathways with the mammalian nephric duct and ureteric lineage [32,42,44,69,70]. Consequently, abnormalities affecting this region can provide informative readouts of disrupted developmental programs that are relevant to human CAKUT pathogenesis. *WNT4* provides a clear example of conservation of nephrogenic signaling pathways across vertebrates. In mammals, *WNT4* is expressed in the metanephric mesenchyme, where it is a key regulator of the mesenchymal-to-epithelial transition required for nephron formation [71,72,73,74]. Similarly, zebrafish studies have demonstrated an essential role for *wnt4* in pronephric tubulogenesis, highlighting the conservation of developmental mechanisms that govern nephrogenesis across species. While the effect of *wnt4* knockout/knockdown on renal development was not directly assessed in zebrafish models, a study found smaller or absent proximal pronephric tubules in zebrafish where the Wnt pathway has been disrupted [75].

Other signaling molecules further highlight the usefulness of zebrafish as a model to study pronephric duct and tubule morphogenesis in the context of CAKUT. *CRKL*, which is known to signal through receptor tyrosine kinases, reveals glomerular cysts, pericardial edema, and reduced pronephric duct length when *crkl* is knocked out or knocked down in zebrafish [76,77,78]. This example highlights how the disruption of an intracellular signaling molecule can impact renal development in zebrafish. Knockdown of *fat4*, a Hippo pathway signaling molecule, reveals perturbed tubule shape due to pronephric cysts [79]. Other genes involved in ureteric bud and collecting duct morphogenesis, such as *RET* and *DSTYK*, should be interpreted carefully. While there is currently no zebrafish phenotype associated with *ret*, it is expressed in the distal late tubule and pronephric duct, suggesting an important function during development for these structures [32]. Similarly, knockdown of *dstyk* in zebrafish results in pericardial edema and abnormal cloacal development, including defective formation of the pronephric opening. These phenotypes were attributed to impaired fibroblast growth factor (FGF) signaling, supporting a conserved role for *DSTYK* in developmental pathways required for urinary tract morphogenesis [59]. Together, these studies demonstrate that even though zebrafish cannot directly model ureteric bud induction and branching, perturbing these signaling pathways can reveal conserved defects in duct extension, cloacal opening, segmentation, and tubule morphogenesis.

### 3.3. Extracellular Matrix & Adhesion Molecules

**Figure 3:**
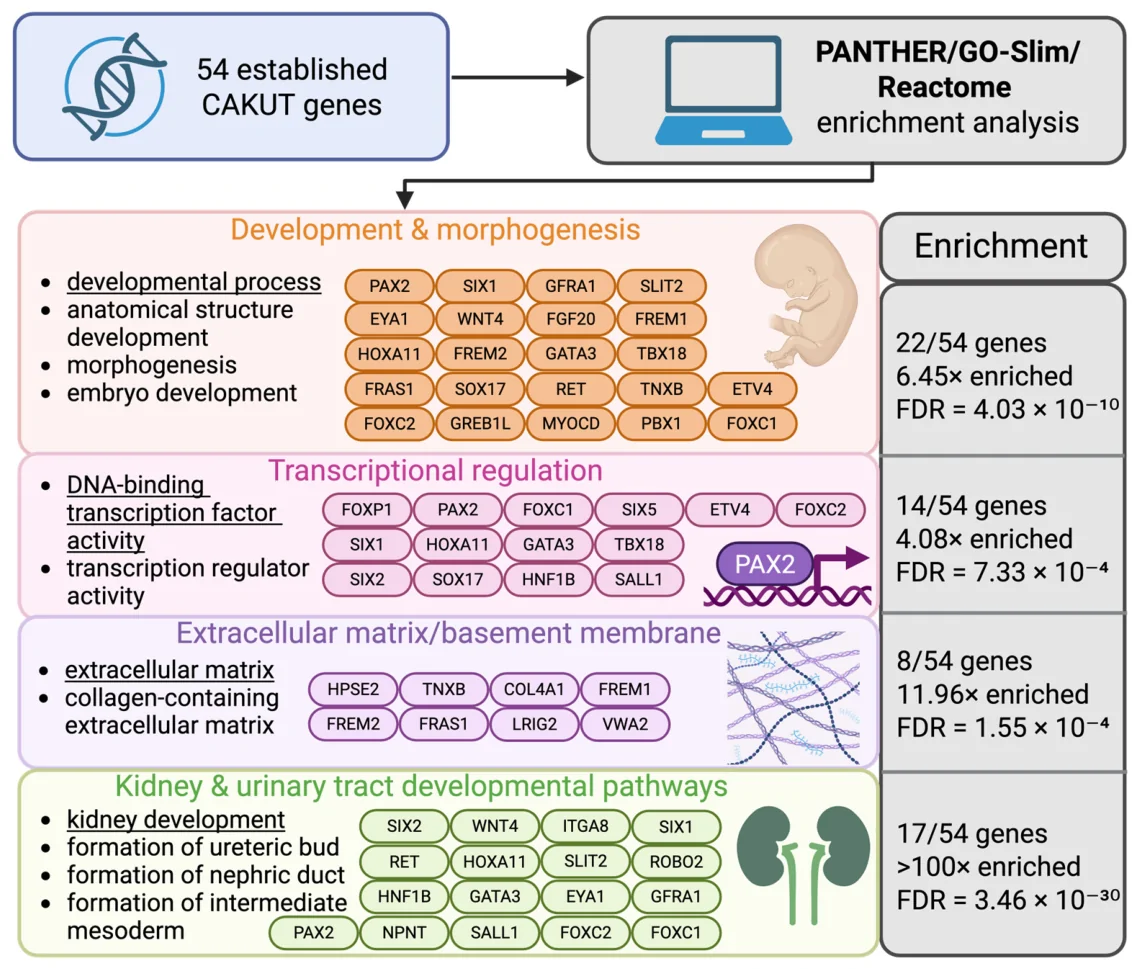
Functional enrichment of established congenital anomalies of the kidney and urinary tract (CAKUT)-associated genes. Functional enrichment analysis of 54 curated high-confidence human CAKUT-associated genes was performed using PANTHER Classification System. Enriched biological processes highlight developmental themes relevant to CAKUT pathogenesis. FDR: false discovery rate. Created in BioRender. Nurcombe, Z. (2026) https://BioRender.com/6qznk6k.

In addition to the extensive list of transcription factors, several established CAKUT-associated genes encode components of the ECM or epithelial adhesion proteins. This highlights the importance of tissue structure and integrity during kidney and urinary tract development. However, ECM and epithelial adhesion proteins serve functions that extend far beyond providing structural support. These molecules act as critical regulators of developmental signaling by organizing the extracellular environment, modulating growth factor availability, and transmitting biochemical and mechanical cues from the extracellular space to intracellular signaling networks. As a result, they play essential roles in coordinating cell fate decisions, tissue morphogenesis, and organ development [80,81]. While several ECM genes are among the established CAKUT-associated genes (e.g., *FRAS1*, *ITGA8*, and *HPSE2*), they are still under-investigated in the zebrafish. *NPNT* is one of the clearest examples of an ECM gene investigated in zebrafish. Researchers demonstrated that morpholino knockdown of *npnta* in zebrafish results in a glomerular filtration defect with pericardial edema [82]. While this finding is not a classical CAKUT malformation, *npnta* was reported to be expressed in the pronephros at 24 hpf, and when knocked down, a glomerular defect is observed where zebrafish exhibit pericardial edema, abnormal glomerular basement membrane pathology, and podocyte marker loss (Table S1) [83]. These findings support conserved renal relevance of this gene. Other CAKUT genes involved in ECM biology are relatively uncharacterized in zebrafish. Other ECM genes, such as *COL4A1, FRAS1*, *FREM1* (*frem1b*), and *FREM2* (*frem2b*) do not yet have a reported renal phenotype in zebrafish, but are expressed in the developing zebrafish pronephros, supporting their critical role in renal development [84,85,86]. This observation is supported by our enrichment analysis, which highlighted extracellular matrix organization and epithelial adhesion as prominent biological processes among CAKUT-associated genes, highlighting the opportunity for future studies to explore their role in kidney morphogenesis (Figure 3).

## 4. Key Insights from Zebrafish Studies of CAKUT

Several observations emerge from the curated CAKUT gene set presented in Table S1. First, transcription factors/regulators are the most frequently assessed functionally in zebrafish. Common defects seen in this gene class are pronephric specification, segmentation, tubule differentiation, and morphogenesis of the distal duct. A common phenotype seen across genes is some sort of perturbation of the distal pronephric duct, a region of the pronephros that has conserved nephric duct epithelial programs [29,32,38,43]. Even though zebrafish do not undergo ureteric bud induction or branching, these phenotypes may still be relevant to understanding the developmental programs and signaling involved in the formation of the ureteric bud and collecting duct system in mammals. In contrast, ECM and epithelial adhesion molecules are comparatively underrepresented among functionally characterized CAKUT genes in zebrafish. Although traditionally viewed as structural components, these proteins also regulate developmental signaling by mediating cell–cell and cell–matrix interactions and by modulating the extracellular environment. Given their established roles in renal morphogenesis and tissue organization, the limited functional characterization of this gene class represents a significant gap in our current understanding of CAKUT biology. Certain CAKUT-associated genes produce phenotypes that closely parallel aspects of the corresponding human phenotype, whereas others require a more nuanced interpretation. For example, CAKUT causing *PAX2* variants in humans commonly result in renal hypodysplasia. In zebrafish, knockdown of *pax2a* results in disturbed pronephric tubule differentiation and distal duct defects, which could be interpreted as a similar phenotype [36]. On the contrary, *NPNT*, which has been associated with severe CAKUT phenotypes including renal agenesis, causes tubular defects and abnormal glomerular filtration phenotypes in zebrafish, which do not fully resemble the more severe human phenotype [56,82,87]. Other genes do not yet have an established zebrafish phenotype, but are expressed in the zebrafish kidney, suggesting that they have developmental relevance. It is important to recognize that the renal phenotypes observed in zebrafish models of CAKUT generally fall into a small, limited number of recurring categories, including defects in pronephric patterning and differentiation, distal pronephric duct morphogenesis, pronephric cyst formation, and glomerular filtration abnormalities. While these phenotypes do not typically correspond directly to specific human CAKUT malformations, they provide informative readouts of disrupted developmental processes. As such, zebrafish models should be viewed primarily as tools for interrogating conserved mechanisms of nephrogenesis, epithelial differentiation, and renal morphogenesis rather than as systems that recapitulate individual human CAKUT phenotypes on a one-to-one basis.

## 5. Experimental Approaches for Modelling CAKUT in Zebrafish

**Figure 4:**
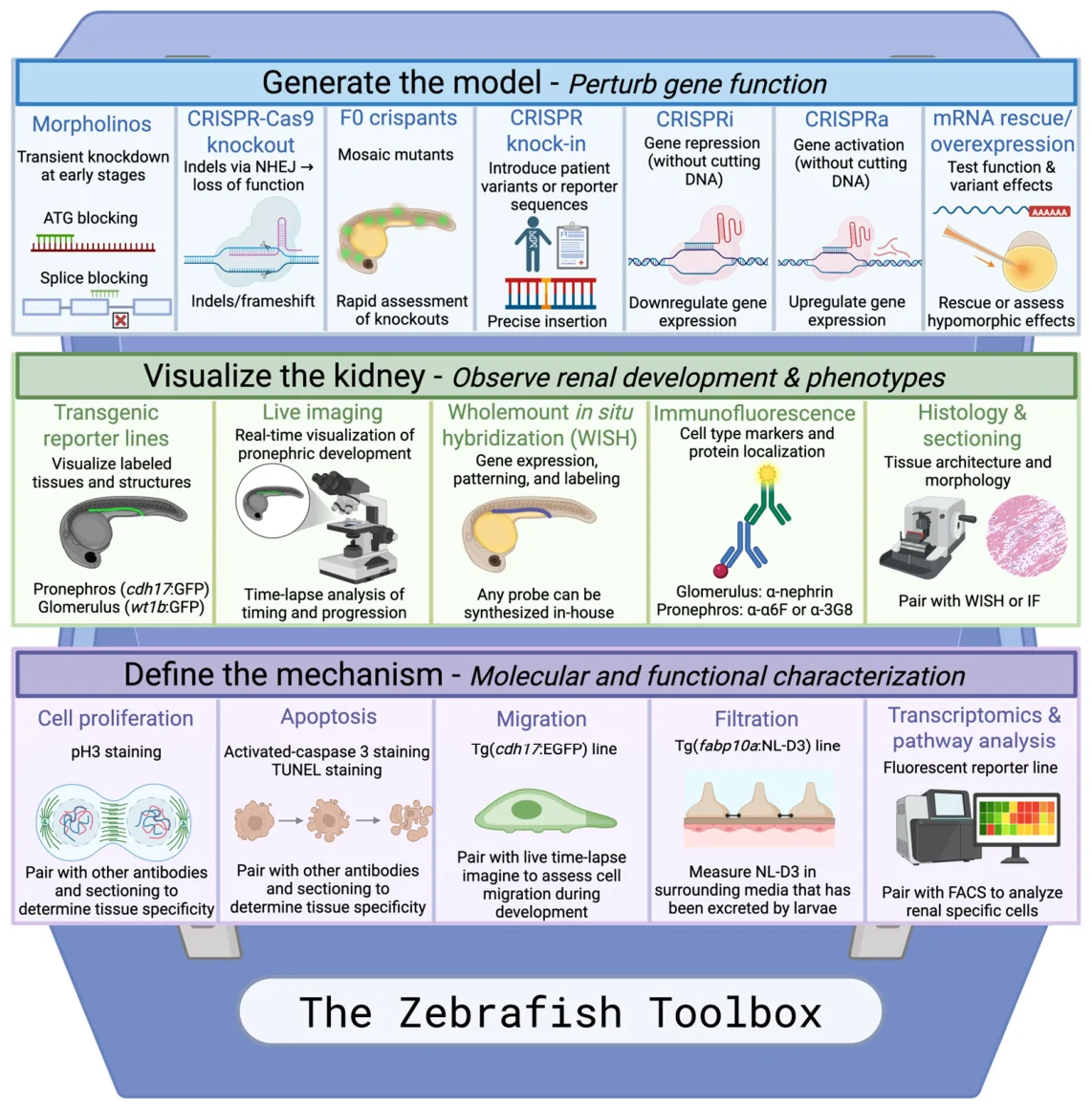
The zebrafish toolbox. Available methods and techniques that can be used to assess gene function and renal development in zebrafish. The toolbox begins with perturbing gene function, visualizing the kidney, and ending with defining the mechanism. IF: immunofluorescence, NEHJ: non-homologous end-joining, WISH: wholemount in situ hybridization. Created in BioRender. Nurcombe, Z. (2026) https://BioRender.com/cq70yk9.

Zebrafish offer several practical advantages as an in vivo model to study kidney development, as well as the clinical relevance of candidate CAKUT genes and VUSs. Their pronephric kidney develops rapidly during embryogenesis, allowing renal phenotypes to be assessed within the first few days of life. Because embryos develop ex utero and are optically transparent, the developing pronephros can be readily visualized and monitored in vivo. In addition, zebrafish are highly amenable to genetic manipulation, enabling efficient gene knockdown, genome editing, transgenic approaches, and functional rescue experiments [27,33,44]. These characteristics allow researchers to assess candidate genes and their variants in vivo with comparative ease with a wide variety of available tools (Figure 4).

### 5.1. Antisense Morpholino Oligonucleotides

One of the most widely used approaches for transient genetic manipulation in zebrafish is the use of antisense morpholino oligonucleotides. Morpholinos are synthetic nucleic acid analogues that inhibit gene function by blocking mRNA translation or interfering with pre-mRNA splicing, thereby enabling rapid assessment of gene function during early embryonic development. Zebrafish embryos are microinjected at the single cell stage with a morpholino oligonucleotide that has been designed to specifically target a gene of interest. There are two types of morpholinos: splice site binding and ATG binding, which inhibit intronic splicing of mRNA, leading to nonsense mediated decay, or sterically hinder the ribosome from binding to the ATG site of the mRNA, resulting in no gene translation [88,89]. Morpholino-mediated knockdown transiently suppresses gene function during early zebrafish development. The efficiency should be validated where possible as the efficacy and duration of suppression can vary based on sequence, dose, and target [89,90].

### 5.2. CRISPR-Cas9 Gene Knockouts

Clustered Regularly Interspaced Short Palindromic Repeats (CRISPR)-Cas9 is the modern genetic validation approach that complements screening with morpholinos where gene and exon specific guide RNA (gRNA) and Cas9 is injected into the zebrafish embryo, which induces double stranded breaks at the target site, leading to repair via non-homologous end joining (NHEJ) which will often produce indels and frameshift mutations, leading to loss of function alleles [91,92]. This method is useful for generating a stable mutant knockout line, but can also be used to generate F0 mutants, or crispants [91,93,94,95]. While timesaving and considered to be relatively efficient, one caveat to the F0 mutants is that they are mosaic knockouts [96]. Both CRISPR mutants and crispant zebrafish are useful tools for candidate CAKUT genes roles in the developing kidney, as demonstrated with zebrafish *six2b* [68] (Table S1).

Following the introduction of CRISPR-Cas9 genome editing, several studies reported discrepancies between phenotypes observed in morpholino-treated embryos (morphants) and those observed in stable genetic mutants [90,97,98]. In a number of cases, CRISPR-generated mutants failed to recapitulate previously described morphant phenotypes. Subsequent work suggested two principal explanations for this phenomenon. First, maternally deposited mRNA can persist during early embryogenesis and partially compensate for loss of zygotic gene function in genetic mutants, thereby masking early developmental phenotypes [99]. Second, complete gene disruption can trigger genetic compensation mechanisms, resulting in transcriptional adaptation and upregulation of related genes that mitigate the effects of the mutation [90,97,98]. In contrast, morpholino-mediated knockdown typically does not elicit these compensatory responses; however, morpholino oligonucleotides can induce off-target effects by activating the p53 pathway [100]. This can be addressed by co-injecting a morpholino targeting p53; however, any phenotype observed after p53 co-injection should be correctly validated using other morpholinos targeting the same gene, comparing to mutant models, dose response, or mRNA rescue [90,98,100,101]. As a result, discrepancies between morphants and mutants do not necessarily invalidate either approach, but instead highlight distinct biological responses to transient knockdown and stable genetic mutation. Consequently, morpholino and CRISPR-based approaches are often complementary, and findings are most robust when supported by multiple independent lines of evidence.

### 5.3. CRISPR Knock-In Models

In contrast to CRISPR knockout models, CRISPR also allows knock-in approaches to introduce patient-specific VUSs or reporter sequences into the zebrafish genome [102,103,104,105]. Though technically challenging compared to CRISPR knockouts, technology is constantly improving increasing the efficiency of this method [104]. Generating these knock-in models is valuable when studying variant-specific effects for known CAKUT genes. By inserting these patient-specific variants into the zebrafish genome, the variant can easily be assessed for renal phenotypes. However, because precise knock-in approaches require efficient template-mediated repair, founder identification, germline transmission, and the generation of a stable knock-in line, they are time-consuming and poorly suited for scalable screening of large numbers of genes [106,107,108]. Instead, knock-in models may be most useful for characterization of priority variants when long-term phenotyping is required.

### 5.4. CRISPRi

CRISPR interference (CRISPRi) utilizes a catalytically inactive, or dead Cas9 (dCas9) protein [109,110]. Like F0 crispants, CRISPRi is a gene knock down method that targets the genome, but it does not induce double stranded breaks. It instead binds to the genomic region and represses transcription [109,110,111]. CRISPRi appears to be less commonly used in zebrafish compared to methods like morpholino knockdown or CRISPR-Cas9 knockout, likely due to technical challenges including variable repression efficiency, repressor optimization and gRNA dosage, and uncertainty regarding the duration of transcriptional repression during development [111,112]. To our knowledge, CRISPRi has not been widely applied to CAKUT gene investigation in zebrafish, but CRISPRi may be useful in the future for testing regulatory variants or enhancer and promoter function [113].

### 5.5. CRISPRa

CRISPR activation (CRISPRa) is a complementary approach to CRISPRi that shares the use of dCas9. The dCas9 is fused to transcriptional activator domains, and can be used to increase expression of the target genes [109,113,114,115,116]. This method has been demonstrated in zebrafish and can be used as a tool to model increased gene dosage or gain-of-function-like effects [117]. Although gain-of-function mechanisms are not well established for most CAKUT genes, CRISPRa may be useful for testing dosage-sensitive candidate genes, or genes located within copy-number gains [6,118,119,120]. However, its current relevance to CAKUT modeling remains largely theoretical as CRISPRa has not yet been widely applied to CAKUT-associated genes in zebrafish.

### 5.6. Transgenic Reporter Lines and Live Imaging

Stable transgenic reporter lines are an important tool used to investigate renal development in zebrafish. Since zebrafish are transparent and develop ex utero, transgenic reporter lines can allow for fluorescent visualization of developing renal structures live in the zebrafish embryo. Several renal-specific transgenic fish have been developed. These fish label different components of the kidney, including the pronephros as well as the glomerulus (podocytes) [31,121]. These transgenic lines allow investigators to assess renal development and function in both live and fixed specimens and can be readily combined with the genetic approaches described above. One particularly useful example is a transgenic zebrafish line expressing NanoLuc-D3 (NL-D3), a secreted luciferase reporter, under the control of the *fabp10a* promoter [85]. Under normal conditions, NL-D3 is retained within the circulation; however, disruption of the glomerular filtration barrier results in urinary loss of the reporter protein. Measurement of NL-D3 activity in the surrounding water therefore provides a sensitive and non-invasive assay of glomerular filtration function in vivo [85].

For CAKUT, transgenic zebrafish offer several advantages. Labeling the kidney lets researchers assess renal phenotypes directly, instead of relying on secondary phenotype-specific readouts such as pericardial edema, body curvature, or survival. With the use of a transgenic line labeling the pronephros, specific pronephric phenotypes can be observed such as tubule length, tubule dilation, cyst formation, and epithelial organization [122,123]. Since these fish can also be imaged live, time-lapse videos can be taken to assess timing and progression of pronephric development, revealing developmental abnormalities. Despite their utility, transgenic reporter lines provide only a partial view of renal development and function and may not capture all aspects of a given phenotype. Consequently, observations made using transgenic lines should be complemented with additional approaches, including whole-mount in situ hybridization (WISH), immunostaining, and histological analyses, to validate findings and provide a more comprehensive assessment of renal development and morphology.

### 5.7. Molecular and Cellular Labeling Approaches

**Figure 5:**
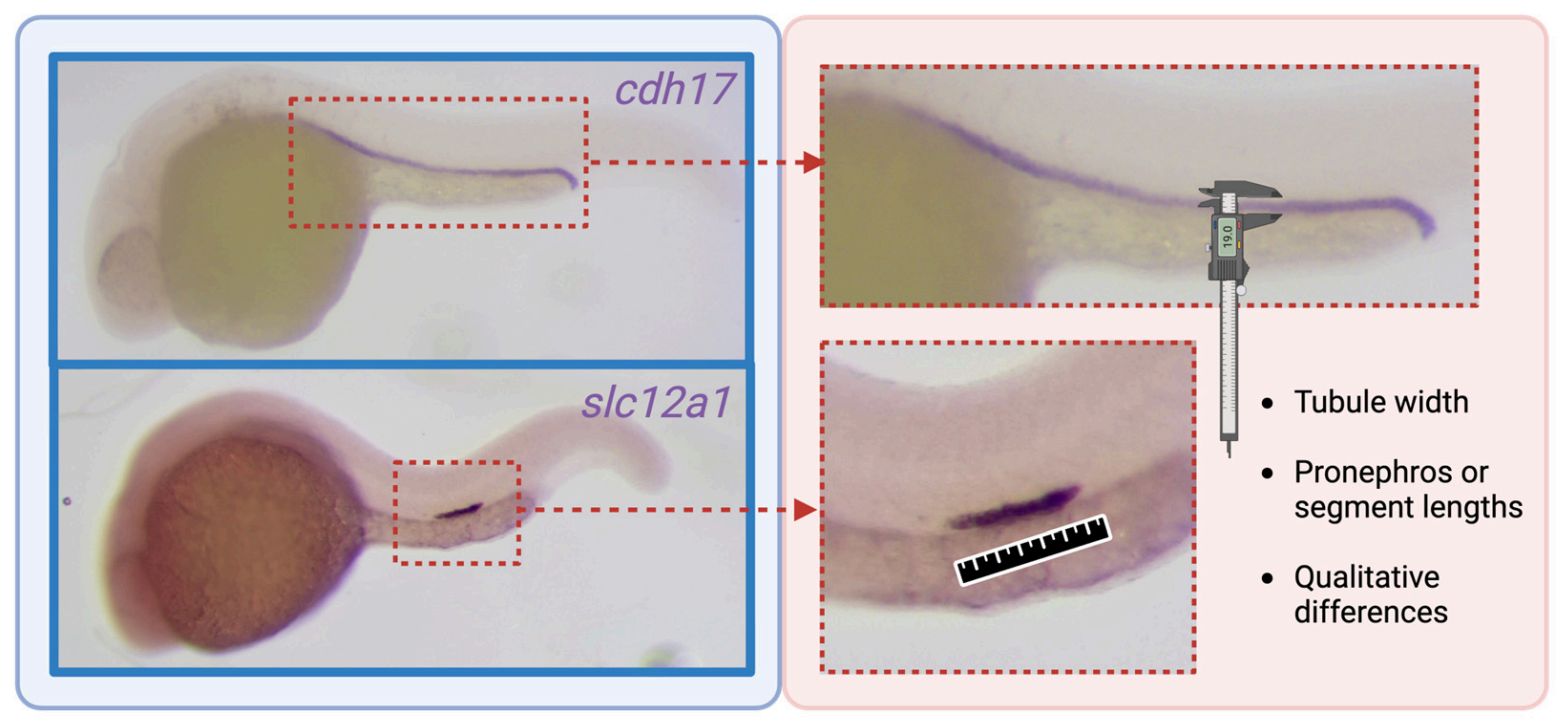
Wholemount in situ hybridization of renal markers and methods to measure changes in renal development. 24 hpf zebrafish are labeled with *cdh17* (pronephros) or *slc12a1* (distal early tubule) antisense probes. After gene perturbation, measurements can be taken with light microscopy images such as the width of the tubule or the length of the pronephros or specific segment. Created in BioRender. Nurcombe, Z. (2026) https://BioRender.com/i7vewof.

In addition to transgenic reporters, other labeling techniques can provide insight into how CAKUT-associated genes contribute to renal development. WISH is a particularly useful labeling technique in which antisense mRNA probes can be synthesized in-house to detect the expression patterns of virtually any gene [124]. This technique enables researchers to readily label the glomerulus, pronephros, and specific nephron segments in fixed zebrafish embryos [32]. For example, an antisense mRNA probe targeting the *cdh17* transcript in zebrafish will exclusively label the full-length pronephros (Figure 5). Other probes targeting genes such as *wt1b*, *slc20a1a*, *trpm7*, *slc12a1*, *stc1*, *slc12a3*, and *gata3* will label the glomerulus (podocytes), proximal convoluted tubule, proximal straight tubule, distal early tubule, corpuscle of Stannius, distal late tubule, and the pronephric duct, respectively [32]. Additional probes can be readily synthesized to meet the specific needs of the researcher. These probes are useful to determine if gene alteration leads to abnormal kidney specification, altered nephron patterning, or disrupted pronephric development, which are all relevant when assessing CAKUT-associated genes.

Additional techniques such as immunolabeling and other related techniques can further define the pathogenic mechanisms underlying manipulated CAKUT genes. A notable limitation of immunostaining in zebrafish is the relatively limited availability of well-validated antibodies that reliably recognize zebrafish antigens. As a result, characterization of specific cell types, proteins, or signaling pathways can be challenging and may require the use of transgenic reporter lines, in situ hybridization, or alternative molecular approaches [125,126]. It is therefore important to validate antibodies before use or to follow an established protocol with already validated antibodies. There are antibodies to assess the development and morphology of the pronephros, such as α-α6F and α-3G8, which label the whole pronephros and the anterior half of the pronephros, respectively [27]. Other antibodies such as α-aPKC label the apical polarity complex, allowing for assessment of cell polarity within the pronephric tubule [70]. In addition to tubule labeling, there are several antibodies that can label the zebrafish glomerulus as well such as α-nephrin and α-podocin [127,128]. In addition to labeling CAKUT-relevant structures, certain cellular processes can be labeled with antibodies as well that may be relevant depending on the gene and CAKUT phenotype. Using α-phospho-histone H3 (α-pH3) will label mitotic cells, useful for measuring increases or decreases in cell proliferation, which are important aspects of embryonic development [129]. Apoptosis can also be assessed with antibody staining for α-activated Caspase 3, or a non-antibody-based method using Terminal deoxynucleotidyl transferase dUTP Nick-End Labeling (TUNEL) [129,130].

### 5.8. Histology

Although the above-mentioned labeling techniques are useful for whole-mount imaging, whole-mount preparations can sometimes obscure tissue-level abnormalities or the precise localization of signals. Sectioning zebrafish embryos can enable more precise visualization of internal structures and cellular architecture. Coronal or sagittal sections facilitate the identification of tubular abnormalities that may not be readily apparent in whole-mount preparations [122]. When combined with labeling techniques such as immunohistochemistry or in situ hybridization, sectioning can further aid the characterization of renal phenotypes, including cyst formation, cell organization, or cell polarity defects [79].

### 5.9. mRNA Rescue and Variant Interpretation

mRNA rescue experiments are crucial in connecting zebrafish phenotypes to human genetics. In a typical rescue experiment, an endogenous gene is first disrupted, often by morpholino knockdown or CRISPR-based mutagenesis, and exogenous mRNA encoding the wildtype gene product is then introduced to determine whether restoration of gene function can rescue the observed phenotype. This approach is not unique to zebrafish, but has been performed in *Xenopus* and *Drosophila* as well [131,132,133,134]. Phenotype rescue with human mRNA has been established in zebrafish and used to investigate the CAKUT gene *GREB1L*, and has been used for other disease genes as well [131,135]. Sanna-Cherchi et al., (2017) successfully rescues a morphant phenotype (reduced proximal convoluted tubule size) by the injection of wildtype human *GREB1L* mRNA and proceed to inject patient-specific *GREB1L* variant mRNA, which result in no phenotypic rescue [131]. Their results suggest that these pathogenic variants do not have the ability to rescue the renal-specific phenotype, unlike the wildtype human protein. This finding is important because this suggests that zebrafish can be used as a tool to validate the pathogenicity of VUSs in known CAKUT genes. As zebrafish develop quickly, evidence pertaining to a VUS could theoretically be obtained in a matter of weeks.

### 5.10. Transcriptomics and Pathway Analysis

By utilizing fluorescence-activated cell sorting (FACS), the fluorescent cells of transgenic zebrafish can be isolated, for downstream bulk RNAseq analysis [136]. With this, relative gene expression and pathway changes can be observed exclusively in the developing kidney, reducing the signal dilution of using the whole embryo [136,137]. Knockdown of CAKUT genes may produce a unique transcriptomic signature compared to wildtype. This signature may reveal altered developmental programs or signaling pathways involved in nephron development, epithelial/tubule differentiation, or pronephric morphogenesis and may also serve as a useful readout for mRNA rescue experiments [38,138]. This signature could then be used to identify candidate CAKUT genes and assess if mRNA rescue can restore the original transcriptomic signature. Generating a disease transcriptomic signature has been demonstrated in kidney organoids modeling glomerulopathies [139,140].

## 6. Interpreting Zebrafish Phenotypes as Developmental Readouts

**Figure 6:**
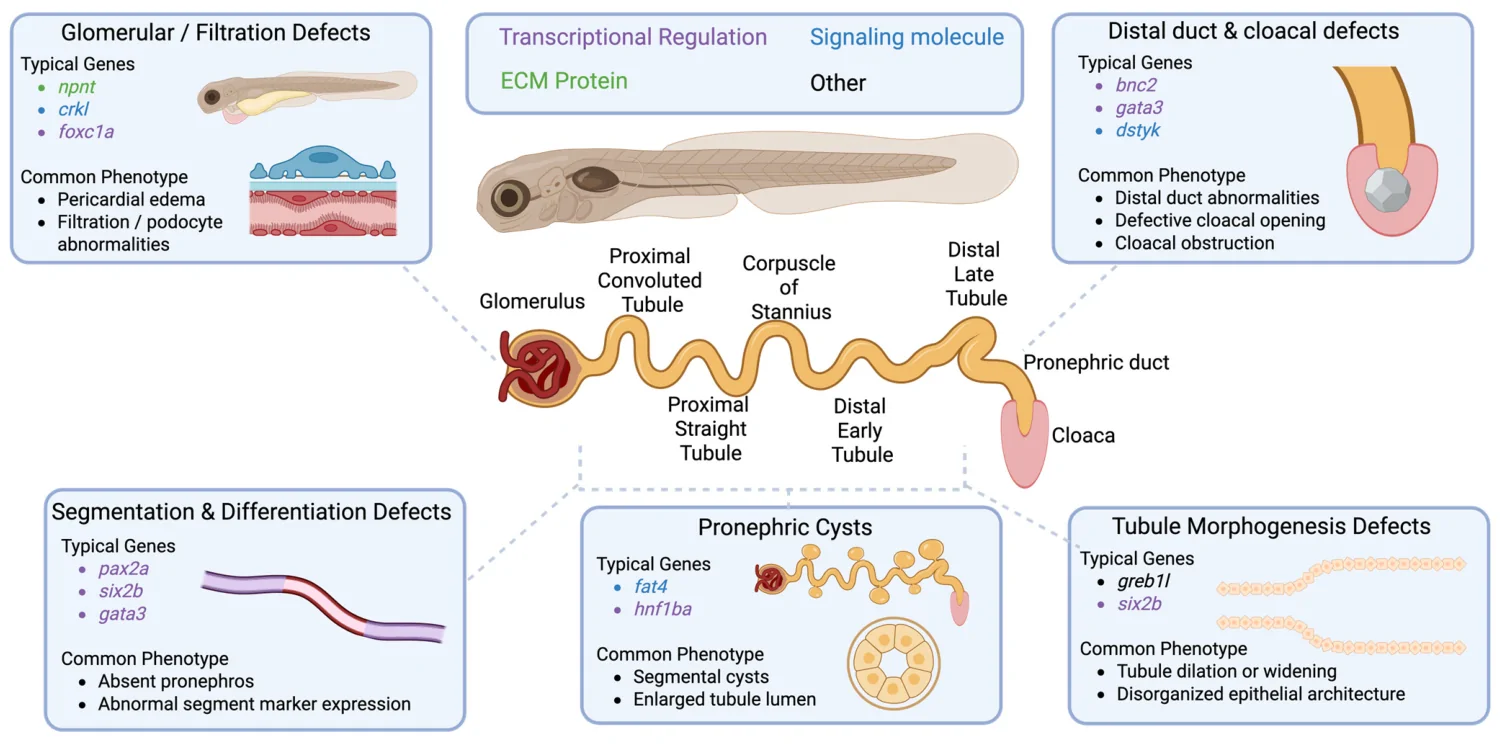
Congenital anomalies of the kidney and urinary tract (CAKUT) phenotypes observed in zebrafish. Several renal phenotypes can be observed in zebrafish in the event of a disrupted CAKUT gene. These range from differentiation, to structural, to glomerular defects. Font colour of the gene reflects its biological role: transcriptional regulation (purple), signaling molecule (blue), extracellular matrix (ECM) protein (green), other (black). Created in BioRender. Nurcombe, Z. (2026) https://BioRender.com/dfdplm8.

While CAKUT encompasses a highly heterogeneous spectrum of anatomical malformations in humans, the corresponding phenotypes observed in zebrafish tend to converge into a smaller number of developmental categories. Rather than recapitulating individual CAKUT phenotypes on a one-to-one basis, zebrafish models most commonly exhibit defects in nephron specification and patterning, distal duct morphogenesis, cyst formation, or glomerular function (Figure 6). Examples of how to measure tubule defects can be seen in Figure 5. This convergence suggests that diverse forms of human CAKUT may arise from disruption of a limited number of conserved developmental pathways. While these phenotypes do not fully recapitulate the anatomical spectrum of human CAKUT, zebrafish should be viewed primarily as an interpretive model for studying the conserved developmental mechanisms that govern kidney and urinary tract morphogenesis. Accordingly, the value of zebrafish models lies not in anatomical equivalence to human disease, but in their ability to reveal how genetic perturbations disrupt developmental pathways relevant to CAKUT pathogenesis.

### 6.1. Patterning and Segmentation: CAKUT Genes Can Disrupt Early Nephron Identity

Several zebrafish models of CAKUT-associated genes show defects in nephron segmentation or segment-specific differentiation, suggesting abnormal nephron development rather than nonspecific tubule malformation. For example, disruption of *hnf1ba/hnf1bb* impairs proximal and distal tubule identity, while *pax2a* loss disrupts pronephric tubule differentiation and patterning [36,46]. This phenotype should be interpreted differently than just a tubule malformation: it suggests a developmental fate specification problem [33,38,46]. As previously discussed in this review, transcription factors are particularly informative as they have roles in early patterning, and some transcription factors reveal these phenotypes such as *pax2a* and *hnf1ba/hnf1bb* [36,46]. These studies suggest that some CAKUT-associated genes impact specific developmental pathways involved in cell fate, rather than resulting in a non-specific renal malformation.

### 6.2. Distal Duct and Cloacal Phenotypes: Interpreting Conserved Developmental Readouts

Because zebrafish kidney development does not involve ureteric bud induction or branching morphogenesis, many CAKUT phenotypes that arise from abnormalities of the metanephric kidney or collecting system cannot be modeled directly. In zebrafish, the distal pronephros comprises distal tubule segments of the pronephric duct. The distal early and distal late segments are generally considered analogous to the mammalian distal nephron segments, and the pronephric duct belongs to the conserved nephric duct lineage and drains to the cloaca [27,33,44]. This distinction is particularly important when investigating candidate CAKUT genes, as the mammalian nephric duct gives rise to the ureteric bud and, ultimately, the collecting system. Consequently, developmental abnormalities involving the distal pronephric duct may provide insight into conserved pathways that regulate nephric duct development and the formation of downstream structures implicated in CAKUT in humans [29,32,33,43,141]. Interestingly, *BNC2* is a CAKUT-associated gene that is known to cause lower urinary tract obstruction in humans (MIM# 618612), and *bnc2* knocked down in zebrafish results in cloacal obstruction [57]. In addition, loss of *gata3*, a CAKUT gene associated with several human metanephric phenotypes, results in distal pronephric duct defects in zebrafish [67,142] (Kitzler laboratory, unpublished observations). Because this structure belongs to the conserved nephric duct lineage and is governed by many of the same developmental pathways that regulate nephric duct and collecting system formation in mammals, phenotypes affecting the distal pronephric duct can provide important insights into mechanisms relevant to human CAKUT. Collectively, these findings suggest that although zebrafish lack a true mammalian lower urinary tract, the distal pronephric duct nevertheless provides a valuable developmental readout for studying genes involved in lower urinary tract development.

### 6.3. Tubule Dilation/Cysts/Epithelial Organization: Morphogenesis, Polarity, and Tissue Integrity

While renal cysts and tubule dilation phenotypes may not directly recapitulate human CAKUT phenotypes, they are still relevant to kidney morphogenesis. For example, *FAT4* is a cause of syndromic CAKUT in the form of Van Maldergem syndrome (MIM# 615546) and is involved in Hippo signaling [64,143,144]. Combined knockdown of *fat1* and *fat4* results in the development of pronephric cysts [79]. This provides an example of CAKUT-associated genes producing a cystic pronephric phenotype in zebrafish, consistent with a role for *fat1/fat4*-associated signaling in renal morphogenesis. Renal cysts and tubule dilation can be investigated further, going beyond more than just a morphological phenotype. Sectioning and staining can reveal disruption of epithelial polarity or lumen maintenance [70].

### 6.4. ECM/Adhesion/Basement Membrane: An Underdeveloped but Important Frontier

Kidney and urinary tract development rely on the structural and signaling roles of the ECM and BM [145]. Currently, zebrafish work is underrepresenting ECM and integrin associated CAKUT genes, and it is therefore difficult to elucidate common phenotype patterns. Importantly, ECM/BM-associated genes may not produce nephron differentiation defects but instead manifest as abnormalities in tubular organization. Because integrin signaling regulates multiple cellular processes, including proliferation, migration, and survival, assessment of these phenotypes provides a valuable opportunity to functionally characterize ECM/BM-associated genes that have not yet been investigated in zebrafish [80,146].

### 6.5. Glomerular Filtration Phenotypes: Useful Readouts, but Look Beyond the Pronephros

Common phenotypes observed in zebrafish are pericardial and periorbital edema, proteinuria, and glomerular abnormalities, all of which are useful indicators of renal dysfunction or developmental abnormalities [68,76,82]. However, these phenotypes should be distinguished from structural CAKUT readouts unless they are accompanied by evidence of abnormal pronephros. This distinction is important because orthologs of human CAKUT-associated genes may produce different phenotypic readouts across model organisms. For example, *NPNT* is associated with renal agenesis in human and mice, but with glomerular phenotypes in the zebrafish [82,87,147]. Accordingly, zebrafish phenotypes may support a role for a gene in renal development or function, but the observed phenotype should be interpreted in the context of species-specific renal anatomy and the experimental assay employed. Moreover, pericardial and periorbital edema are secondary phenotypic readouts that may reflect a compromised glomerular filtration barrier despite apparently normal developed pronephros by light microscopy, with abnormalities detectable only at the ultrastructural level.

### 6.6. Successful Phenotype Identification

Identification of a renal-specific phenotype will elucidate a CAKUT-relevant developmental mechanism in the zebrafish. Once a reproducible and recognizable phenotype has been identified and appropriately interpreted, they become useful for gene validation and variant interpretation. This can be done using the previously discussed mRNA rescue of patient-specific variants or CRISPR knock-ins.

## 7. Clinical Translation of Zebrafish CAKUT Models

### 7.1. Clinical Value: Functional Prioritization of Gene Candidates

Clinical sequencing identifies candidate CAKUT genes and VUSs, but it can be difficult for clinicians to know which genes and variants are biologically relevant [5,17,131]. Zebrafish models offer an approach to solve this dilemma. Using zebrafish can contribute to functional prioritization by showing whether a gene or variant affects developmental processes relevant to CAKUT pathogenesis.

### 7.2. Variant Interpretation: Zebrafish as Functional Evidence, Not Stand-Alone Proof

The phenotype data collected from zebrafish can be used as functional evidence to support variant interpretation for ClinGen and American College of Medical Genetics (ACMG) classification [20,148]. The most useful variant information derived from zebrafish models is by testing patient-specific variants directly for their rescue ability. Patient mRNA may rescue, fail to rescue, or partially rescue a renal developmental phenotype, suggesting a specific variant is either benign, pathogenic, or hypomorphic, respectively. Functional data obtained from zebrafish studies should not be interpreted in isolation or viewed as definitive evidence of pathogenicity. Rather, these findings are most informative when integrated with complementary sources of evidence, including clinical phenotyping, segregation data, population frequency data, computational predictions, and other functional studies. In this context, zebrafish models can provide valuable biological evidence regarding gene function and variant impact, helping to clarify the relevance of candidate variants and contributing to the interpretation of variants of uncertain significance.

### 7.3. Integration with Human Kidney Organoid Platforms

While zebrafish serve as a rapid in vivo model to investigate candidate CAKUT genes and VUSs, human kidney organoids can provide a useful tool to perform follow-up validation [24,149]. Kidney organoids that are derived from human pluripotent stem cells develop nephron-like structures and kidney cell lineages that make them useful for studying nephrogenesis and renal cell differentiation [149]. Human kidney organoids can be combined with gene editing to model disease-relevant cellular phenotypes and test different candidate CAKUT genes or variants in a human developmental system [150,151]. By using zebrafish as a rapid first-pass in vivo model to prioritize candidate genes and collect functional data, organoids can then be used to determine whether the same gene or variant disrupts renal cell specification, epithelial organization, nephron differentiation, or signaling pathways [24,149]. While human kidney organoids provide an exciting and useful developmental in vitro model, they have their own set of limitations. This includes an incomplete maturation, limited vascularization, and incomplete modeling of whole-organ anatomy as well as lack of urinary drainage [152,153]. With these considerations, zebrafish and kidney organoids can be used as complementary systems that can strengthen functional evidence when used together.

### 7.4. Future Direction: A Scalable Preclinical Bridge

**Figure 7:**
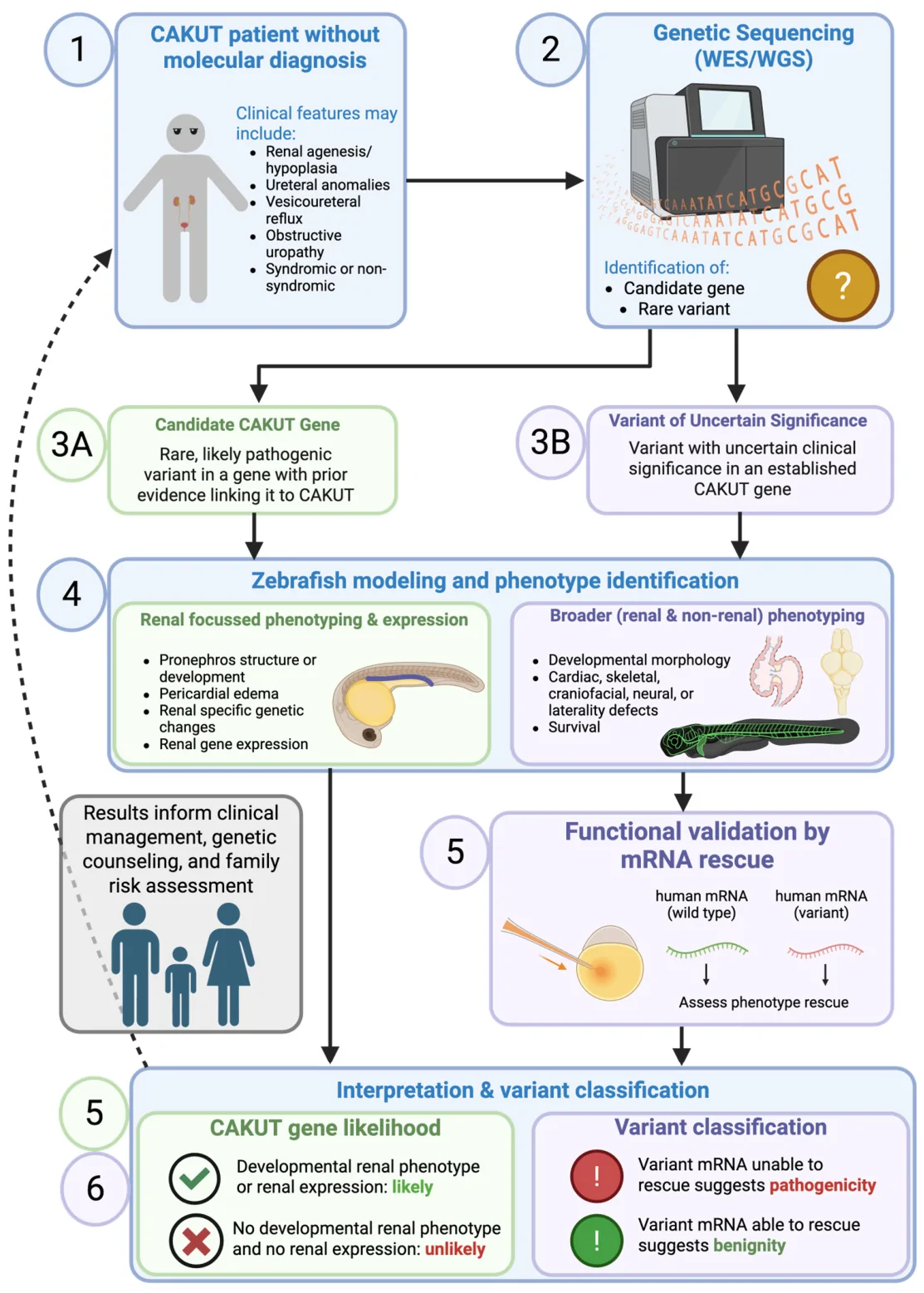
Zebrafish-based workflow for functional investigation of congenital anomalies of the kidney and urinary tract (CAKUT)-associated genes and variants. Patients with a CAKUT phenotype without a molecular diagnosis may undergo genomic sequencing that identifies either a candidate CAKUT gene or variant of uncertain significance (VUS). Candidate genes can be tested in zebrafish to determine if they have a role in renal development. Zebrafish can be used to rapidly gather evidence towards VUS classification. The functional evidence acquired with zebrafish can support clinical interpretation, genetic counseling, and family risk assessment. Blue: steps required for both candidate gene assessment and VUS validation. Green: steps unique to candidate gene assessment. Purple: steps unique to VUS validation. WES: whole-exome sequencing, WGS: whole-genome sequencing. Created in BioRender. Nurcombe, Z. (2026) https://BioRender.com/vy3sbt8.

As genomic sequencing becomes increasingly accessible in both research and clinical settings, the number of candidate genes and variants identified in individuals with CAKUT is expected to grow substantially. However, the ability to functionally interpret these findings has not kept pace with the rate of gene discovery. This challenge is particularly pronounced for developmental genes, whose functions are often context-dependent and require evaluation within the spatial and temporal framework of organogenesis. As a result, the lack of scalable and biologically relevant functional assays is emerging as a major bottleneck in the field, limiting our ability to establish gene-disease relationships, resolve variants of uncertain significance, and translate genomic discoveries into biological and clinical insights. Zebrafish offer a fast, relatively easy, and inexpensive model system to prioritize variants for further investigation by using more laborious and more costly models such as patient-specific organoids, mice, and extensive family studies. Laboratories should develop pipelines for phenotype identification that could utilize transgenic zebrafish, morpholino or crispant phenotype screening, and patient-specific mRNA rescue to generate fast, reproducible functional evidence to help prioritize candidate CAKUT genes for further study and classify novel VUSs (Figure 7).

## 8. Conclusions

Zebrafish have emerged as a valuable model system for investigating the developmental mechanisms underlying CAKUT. Although the zebrafish kidney is anatomically simpler than the mammalian metanephros, it shares many conserved genetic pathways and developmental programs that are directly relevant to kidney and urinary tract development. Consequently, zebrafish models should not be viewed as direct anatomical replicas of human CAKUT, but rather as powerful systems for interrogating the conserved developmental processes that underlie disease pathogenesis. As next-generation sequencing continues to advance and more candidate CAKUT genes and variants are uncovered, functional validation will remain a major bottleneck in CAKUT research and clinical gene interpretation. Zebrafish have the potential to bridge this gap by linking gene disruption or variant expression to developmental phenotypes and renal morphology. When combined with human genetic and phenotype data, mammalian models, and cell-based assays, zebrafish studies can strengthen evidence for disease causality and help clarify developmental mechanisms underlying CAKUT. In the future, national and international collaborative centers that integrate next-generation sequencing, deep clinical phenotyping, variant curation, and functional validation could accelerate the process of interpreting CAKUT-associated genes. Such centralized efforts would be valuable for rare developmental kidney diseases, where individual centers may only identify a small number of patients, but shared pipelines could support higher-throughput discovery and validation using complementary models such as zebrafish, kidney organoids, and mammalian systems. Moving forward, the greatest value of zebrafish may lie not in replacing other model systems, but in serving as a rapid and complementary platform for gene discovery, variant interpretation, and mechanistic insight in CAKUT.

## 9. Materials and Methods

This article was prepared as a narrative review and incorporates curated summary tables of the available literature. It was not designed or conducted as a systematic review or meta-analysis; therefore, formal PRISMA-based study screening, risk-of-bias assessment, and quantitative synthesis were not performed.

### 9.1. Literature Review and Source Verification

A narrative literature review was conducted to identify studies relevant to CAKUT genetics, vertebrate kidney development, zebrafish pronephros development, zebrafish models of CAKUT-associated genes, and experimental approaches for functional gene and variant interpretation. Literature searches were performed using PubMed/MEDLINE and Google Scholar. Search terms included combinations of “CAKUT,” “congenital anomalies of the kidney and urinary tract,” “zebrafish,” “pronephros,” “kidney development,” “renal development,” “nephric duct,” “ureteric bud,” “vesicoureteral reflux,” “renal agenesis,” “renal dysplasia,” “hydronephrosis,” “morpholino,” “CRISPR,” and individual CAKUT-associated gene names.

Primary research articles were prioritized where possible, particularly for statements related to developmental mechanisms, gene function, zebrafish renal phenotypes, and animal model data. Review articles were used to provide clinical or developmental background and to identify additional primary literature. Studies were included if they addressed CAKUT genetics, kidney or urinary tract development, zebrafish pronephros development, functional modeling of CAKUT-associated genes, or methods relevant to gene and variant interpretation. Studies were excluded if they did not address kidney or urinary tract development, lacked interpretable renal phenotypes, or described zebrafish gene function only in unrelated developmental contexts.

AI-assisted tools, including ChatGPT GPT-5.5 Thinking, were used to support literature discovery, refine search terms, identify potentially relevant studies, and clarify database-derived annotations. All sources identified through AI-assisted searches were manually verified by direct examination of the original publications before inclusion. AI-assisted tools were not used as independent sources of evidence, and claims were cited only when supported by the original publication, database entry, or curated resource.

### 9.2. Generation of Curated Gene Table (Table S1)

#### 9.2.1. Gene Inclusion and Source List

The initial gene list was derived from Kolvenbach et al., (2023) and restricted to genes categorized as having human-level evidence for CAKUT [5]. Inheritance patterns were recorded as reported by Kolvenbach et al., (2023) [5].

#### 9.2.2. Human Disease Annotation

Cytoband location and associated OMIM (www.omim.org, accessed on 24 May 2026) phenotypes were curated from OMIM. When multiple OMIM phenotypes were associated with a gene, all phenotypes relevant to renal or urinary tract disease were recorded, while non-renal syndromic features were retained when they provided relevant clinical context.

#### 9.2.3. Zebrafish Orthology and Renal Expression

Zebrafish orthologs were identified using ZFIN (www.zfin.org, accessed on 1 May 2026). Zebrafish renal expression was initially assessed through ZFIN expression records and then verified by reviewing the original cited source when available. When renal expression was explicitly described in the source, the gene was recorded as having reported renal expression. When renal expression was visible in the published expression pattern but not specifically described by the authors, expression was annotated as inferred. When ZFIN listed renal expression without an associated primary source, this was noted as ZFIN-curated expression.

#### 9.2.4. Zebrafish Renal Phenotype

Zebrafish renal phenotypes were identified using ZFIN phenotype annotations, targeted literature searches, and AI-assisted literature discovery. Candidate studies were manually reviewed to confirm whether the reported phenotype involved the pronephros, renal tubules, nephric duct, glomerulus, cloaca, edema, or another kidney/urinary tract-relevant structure. Phenotypes were recorded only when the original publication supported a renal or urinary tract interpretation.

#### 9.2.5. Human Adult Kidney and Bladder Expression

Human kidney expression data were obtained from the Human Protein Atlas (www.proteinatlas.org/, accessed on 24 May 2026). These data are primarily derived from adult tissues and therefore may not fully capture spatiotemporal gene expression patterns during kidney development, when CAKUT phenotypes arise. Although adult expression data do not capture the developmental timing of CAKUT pathogenesis, they were included as supportive context for tissue and nephron-comparative relevance.

For kidney and bladder expression summaries, the top three enriched tissues or cell types were recorded based on the available expression metric. When two tissues or cell types were similarly ranked for the final position, four entries were recorded. Genes with relatively uniform expression across multiple tissues or cell types were annotated as broadly expressed.

Expression levels were categorized as very high (>1000 normalized counts per million [nCPM]) high (1000–100 nCPM), moderate (10–100 nCPM), low (1–10 nCPM), or negligible (<1 nCPM) based on Human Protein Atlas single-cell RNA data.

#### 9.2.6. Embryonic Human Kidney Expression

Embryonic human kidney expression was assessed using the Humphrey 17-week fetal kidney single-cell expression database (www.humphreyslab.com/SingleCell/, accessed on 24 May 2026) [154]. For each gene, the top three enriched cell populations were recorded based on dot plot visualization, prioritizing larger dot size as an indicator of the proportion of expressing cells. When multiple cell populations appeared similarly enriched, up to four populations were recorded. Genes with comparable expression across many populations were annotated as broadly expressed.

#### 9.2.7. Pathway/Function Annotation

For each gene, pathway/function annotations were first reviewed using Kyoto Encyclopedia of Gene and Genomes (KEGG; www.genome.jp/kegg/, accessed on 24 May 2026), Reactome (www.reactome.org/, accessed on 24 May 2026), and UniProt (www.uniprot.org/, accessed on 24 May 2026). When database entries were absent, overly broad, or inconsistent, annotations were manually interpreted in the context of the gene’s known molecular function, published literature, and relevance to kidney or urinary tract development. AI-assisted discussion was used to compare database annotations, clarify ambiguous entries, and support preliminary categorization; however, final pathway assignments were manually reviewed and based on the cited databases and/or primary literature.

#### 9.2.8. Animal Models

Animal model phenotypes in Table S1 were identified through targeted literature searches and AI-assisted literature discovery, followed by manual review of the original publications. Mouse models were prioritized when available, followed by other vertebrate models relevant to kidney or urinary tract development. Only phenotypes supported by the original source were included.

### 9.3. Functional Enrichment Analysis of Established CAKUT Genes

To identify major biological themes represented among established CAKUT-associated genes, functional enrichment analysis was performed using the PANTHER Classification System [155]. A list of 54 curated high-confidence CAKUT-associated genes was submitted using human gene symbols or UniProt identifiers where required to resolve ambiguous gene mapping. Homo sapiens was selected as the organism, and the Homo sapiens reference gene list was used as the background. Over-representation testing was performed using Fisher’s exact test with false discovery rate correction for multiple testing.

Enrichment was assessed using PANTHER Gene Ontology (GO)-Slim Biological Process, PANTHER GO-Slim Molecular Function, PANTHER GO-Slim Cellular Component, PANTHER Protein Class, and Reactome pathway annotations (https://pantherdb.org/, accessed on 2 June 2026). Enriched categories were ranked according to false discovery rate and fold enrichment. Because several Gene Ontology terms were overlapping or hierarchical, representative enriched categories were manually grouped into broader biological themes for interpretation and figure generation. These included developmental processes and morphogenesis, transcriptional regulation, extracellular matrix/basement membrane biology, and kidney and urinary tract developmental pathways. Genes annotated to representative enriched categories were retrieved from the PANTHER results output and used to assign genes to the corresponding figure categories. Genes were allowed to appear in more than one category when annotated to multiple enriched terms.

## Figures and Tables

**Table 1:** Representative congenital anomalies of the kidney and urinary tract (CAKUT)-associated genes and corresponding zebrafish renal phenotypes. Phenotypes are arranged by most severe (red) to least severe (green).

CAKUT Phenotype [52]	Zebrafish Readout(s)	Gene(s) Tested in Zebrafish
**Renal agenesis**	No direct zebrafish equivalent Early pronephric specification, tubule formation, and duct development can be assessed as developmental proxies Glomerular/renal function-associated readouts may be relevant for genes affecting nephron endowment	*GATA3* [53,54]; *GREB1L* [53,55]; *NPNT* [56]
**Posterior urethral valves**	Not studied/not represented in current zebrafish renal phenotype table	Not studied
**Congenital lower urinary tract obstruction/obstructive uropathy**	Pronephric outlet obstruction Cloacal dilatation/cloacal malformation Distal pronephric duct defects Pronephric duct/tubule dilation or widening Pericardial edema as a secondary renal/flow-associated readout	*BNC2* [57,58]
**Ureteropelvic/ureterovesical junction obstruction**	No direct ureteropelvic or ureterovesical junction equivalent in zebrafish Partial proxy readouts include distal pronephric duct/outlet morphology, pronephric drainage defects, duct/tubule dilation, and edema	*DSTYK* [59]; *PAX2* [60]
**Hydronephrosis/impaired urinary drainage**	Pronephric duct/tubule dilation or widening Distal outlet obstruction Cloacal malformation Pericardial edema as a secondary renal/flow-associated readout	*BNC2* [57,58]; *DSTYK* [59]; *GATA3* [53,54]; *HNF1B* [54]; *PAX2* [60]
**Renal hypoplasia/renal hypodysplasia/renal dysplasia**	Reduced/abnormal pronephric duct morphology Impaired pronephric duct/tubule differentiation Reduced tubule length Proximal convoluted tubule swelling or proximal tubule morphogenesis defects Perturbed tubule development Distal pronephric duct defects	*CRKL* [61]; *DSTYK* [59]; *GATA3* [53,54]; *GREB1L* [53,55]; *HNF1B* [54]; *PAX2* [60]; *SIX2* [62,63]; *WNT4* [61]
**Multicystic dysplastic kidney**	Pronephric cyst formation Glomerular cysts Pericardial edema as a renal function/flow-associated readout Abnormal pronephric tubule morphology	*FAT4* [36,64]; *GATA3* [53,54]; *HNF1B* [54]; *NPNT* [56]; *PAX2* [60]; *SIX2* [62,63]
**Syndromic CAKUT/broad or unspecified renal malformation**	Abnormal pronephric tubule or duct development Glomerular filtration defect/proteinuria where reported Podocyte or glomerular basement membrane abnormalities where reported Pericardial edema as a renal function-associated readout	*CRKL* [61]; *FAT4* [36,64]
**Ureterocele/megaureter**	No direct zebrafish equivalent	*GATA3* [53,54]
**Vesicoureteral reflux**	No direct zebrafish equivalent	*DSTYK* [59]; *GATA3* [53,54]; *PAX2* [60]; *SIX2* [62,63]
**Duplex collecting system/duplicated ureter**	No direct zebrafish equivalent	*GATA3* [53,54]; *PAX2* [60]
**Ectopic kidney**	Not studied/not represented in current zebrafish renal phenotype table	Not studied
**Horseshoe kidney**	No direct zebrafish equivalent	*HNF1B* [54]; *PAX2* [60]

## Data Availability

No new data were created or analyzed in this study. Data sharing is not applicable to this article.

## Supplementary Materials

The following supporting information can be downloaded at https://www.mdpi.com/2073-4425/17/8/867/s1: Table S1: Comprehensive list of human CAKUT-associated genes and reported renal phenotypes across model systems. Table S1 references: *ACE* [156,157], *AGT* [158,159], *AGTR1* [158,160], *ARHGEF6* [161], *BNC2* [57,162], *CHRM3* [163], *CHRNA3* [164,165,166], *COL4A1* [85,86,167,168,169,170,171], *CRKL* [76,78,172], *DSTYK* [59,169], *ETV4* [173,174,175], *EYA1* [176,177,178,179,180], *FAM58A, FAT4* [64,79,181], *FGF20* [182,183,184,185,186], *FOXC1* [69,187,188,189,190,191,192,193], *FOXC2* [188,189,194], *FOXP1* [195,196,197,198], *FRAS1* [84,199,200], *FREM1* [199,201], *FREM2* [84,202], *GATA3* [32,43,67], *GFRA1* [203,204,205,206], *GREB1L* [53,131,207], *GRIP1* [84,208], *HNF1B* [38,46,65,66,184,209,210], *HOXA11* [211,212], *HPSE2* [213,214], *ITGA8* [215,216], *LRIG2* [214], *MUC1* [217,218], *MYOCD* [219], *NPNT* [82,83,87,147], *NRIP1* [220], *PAX2* [36,38,221,222], *PBX1* [223,224], *REN* [225,226,227,228], *RET* [32,229,230,231], *ROBO1* [232,233,234], *ROBO2* [233,235,236,237], *SALL1* [32,238,239], *SIX1* [179,240,241], *SIX2* [62,68,242], *SIX5* [243], *SLIT2* [233,234,244], *SOX17* [245], *SRGAP1* [233], *TBX18* [246], *TNXB, TRAP1* [19,247], *UPK3A* [248], *VWA2* [249,250], *WNT4* [71,72,251,252,253], *ZMYM2* [254,255].

## References

[1] Chesnaye N., Bonthuis M., Schaefer F., Groothoff J.W., Verrina E., Heaf J.G., Jankauskiene A., Lukosiene V., Molchanova E.A., Mota C. (2014). Demographics of Paediatric Renal Replacement Therapy in Europe: A Report of the ESPN/ERA–EDTA Registry. Pediatr. Nephrol..

[2] Yerkes E., Nishimura H., Miyazaki Y., Tsuchida S., Brock J.W., Ichikawa I. (1998). Role of Angiotensin in the Congenital Anomalies of the Kidney and Urinary Tract in the Mouse and the Human. Kidney Int..

[3] Woolf A.S. (2022). The Term CAKUT Has Outlived Its Usefulness: The Case for the Prosecution. Pediatr. Nephrol..

[4] Ichikawa I., Kuwayama F., Pope J.C., Stephens F.D., Miyazaki Y. (2002). Paradigm Shift from Classic Anatomic Theories to Contemporary Cell Biological Views of CAKUT. Kidney Int..

[5] Kolvenbach C.M., Shril S., Hildebrandt F. (2023). The Genetics and Pathogenesis of CAKUT. Nat. Rev. Nephrol..

[6] Sanna-Cherchi S., Westland R., Ghiggeri G.M., Gharavi A.G. (2018). Genetic Basis of Human Congenital Anomalies of the Kidney and Urinary Tract. J. Clin. Investig..

[7] Vivante A., Hildebrandt F. (2016). Exome Sequencing Frequently Reveals the Cause of Early-Onset Chronic Kidney Disease. Nat. Rev. Nephrol..

[8] Murugapoopathy V., Gupta I.R. (2020). A Primer on Congenital Anomalies of the Kidneys and Urinary Tracts (CAKUT). Clin. J. Am. Soc. Nephrol..

[9] van der Ven A.T., Vivante A., Hildebrandt F. (2018). Novel Insights into the Pathogenesis of Monogenic Congenital Anomalies of the Kidney and Urinary Tract. J. Am. Soc. Nephrol..

[10] Nicolaou N., Renkema K.Y., Bongers E.M.H.F., Giles R.H., Knoers N.V.A.M. (2015). Genetic, Environmental, and Epigenetic Factors Involved in CAKUT. Nat. Rev. Nephrol..

[11] Vendrig L.M., ten Hoor M.A.C., König B.H., Lekkerkerker I., Renkema K.Y., Schreuder M.F., van der Zanden L.F.M., van Eerde A.M., Groen in’t Woud S., Mulder J. (2025). Translational Strategies to Uncover the Etiology of Congenital Anomalies of the Kidney and Urinary Tract. Pediatr. Nephrol..

[12] Knoers N.V.A.M. (2022). The Term CAKUT Has Outlived Its Usefulness: The Case for the Defense. Pediatr. Nephrol..

[13] Clissold R.L., Hamilton A.J., Hattersley A.T., Ellard S., Bingham C. (2015). HNF1B-Associated Renal and Extra-Renal Disease-an Expanding Clinical Spectrum. Nat. Rev. Nephrol..

[14] Heidet L., Decramer S., Pawtowski A., Morinière V., Bandin F., Knebelmann B., Lebre A.-S., Faguer S., Guigonis V., Antignac C. (2010). Spectrum of HNF1B Mutations in a Large Cohort of Patients Who Harbor Renal Diseases. Clin. J. Am. Soc. Nephrol..

[15] Rasmussen M., Nielsen M.L., Manak J.R., Mogensen H., Lildballe D.L. (2021). PAX2 Variant Associated with Bilateral Kidney Agenesis and Broad Intrafamilial Disease Variability. Clin. Kidney J..

[16] Negrisolo S., Benetti E. (2023). PAX2 and CAKUT Phenotypes: Report on Two New Variants and a Review of Mutations from the Leiden Open Variation Database. Int. J. Mol. Sci..

[17] Rivera-Munoz E.A., Zhao X.E., Rosenfeld J.A., Luna P.N., Shaw C.A., Posey J.E., Scott D.A. (2025). Clinical Exome Sequencing Efficacy and Phenotypic Expansions Involving Non-Isolated Congenital Anomalies of Kidney and Urinary Tract (CAKUT+). Eur. J. Hum. Genet..

[18] Yosypiv I.V. (2012). Congenital Anomalies of the Kidney and Urinary Tract: A Genetic Disorder?. Int. J. Nephrol..

[19] Saisawat P., Tasic V., Vega-Warner V., Kehinde E.O., Günther B., Airik R., Innis J.W., Hoskins B.E., Hoefele J., Otto E.A. (2012). Identification of Two Novel CAKUT-Causing Genes by Massively Parallel Exon Resequencing of Candidate Genes in Patients with Unilateral Renal Agenesis. Kidney Int..

[20] Brnich S.E., Abou Tayoun A.N., Couch F.J., Cutting G.R., Greenblatt M.S., Heinen C.D., Kanavy D.M., Luo X., McNulty S.M., Starita L.M. (2019). Recommendations for Application of the Functional Evidence PS3/BS3 Criterion Using the ACMG/AMP Sequence Variant Interpretation Framework. Genome Med..

[21] Boettcher S., Simons M. (2022). Model Organisms for Functional Validation in Genetic Renal Disease. Med. Genet..

[22] Walawender L., Becknell B., Matsell D.G. (2023). Congenital Anomalies of the Kidney and Urinary Tract: Defining Risk Factors of Disease Progression and Determinants of Outcomes. Pediatr. Nephrol..

[23] van de Hoek G., Nicolaou N., Giles R.H., Knoers N.V.A.M., Renkema K.Y., Bongers E.M.H.F. (2015). Functional Models for Congenital Anomalies of the Kidney and Urinary Tract. Nephron.

[24] Gehrig J., Pandey G., Westhoff J.H. (2018). Zebrafish as a Model for Drug Screening in Genetic Kidney Diseases. Front. Pediatr..

[25] McKee R.A., Wingert R.A. (2015). Zebrafish Renal Pathology: Emerging Models of Acute Kidney Injury. Curr. Pathobiol. Rep..

[26] Nguyen T.K., Petrikas M., Chambers B.E., Wingert R.A. (2023). Principles of Zebrafish Nephron Segment Development. J. Dev. Biol..

[27] Drummond I.A., Majumdar A., Hentschel H., Elger M., Solnica-Krezel L., Schier A.F., Neuhauss S.C.F., Stemple D.L., Zwartkruis F., Rangini Z. (1998). Early Development of the Zebrafish Pronephros and Analysis of Mutations Affecting Pronephric Function. Development.

[28] Dressler G.R. (2006). The Cellular Basis of Kidney Development. Annu. Rev. Cell Dev. Biol..

[29] Costantini F., Kopan R. (2010). Patterning a Complex Organ: Branching Morphogenesis and Nephron Segmentation in Kidney Development. Dev. Cell.

[30] Little M.H., McMahon A.P. (2012). Mammalian Kidney Development: Principles, Progress, and Projections. Cold Spring Harb. Perspect. Biol..

[31] Diep C.Q., Ma D., Deo R.C., Holm T.M., Naylor R.W., Arora N., Wingert R.A., Bollig F., Djordjevic G., Lichman B. (2011). Identification of Adult Nephron Progenitors Capable of Kidney Regeneration in Zebrafish. Nature.

[32] Wingert R.A., Selleck R., Yu J., Song H.-D., Chen Z., Song A., Zhou Y., Thisse B., Thisse C., McMahon A.P. (2007). The Cdx Genes and Retinoic Acid Control the Positioning and Segmentation of the Zebrafish Pronephros. PLoS Genet..

[33] Wingert R.A., Davidson A.J. (2008). The Zebrafish Pronephros: A Model to Study Nephron Segmentation. Kidney Int..

[34] Bouchard M., Souabni A., Mandler M., Neubüser A., Busslinger M. (2002). Nephric Lineage Specification by Pax2 and Pax8. Genes Dev..

[35] Pfeffer P.L., Gerster T., Lun K., Brand M., Busslinger M. (1998). Characterization of Three Novel Members of the Zebrafish Pax2/5/8 Family: Dependency of Pax5 and Pax8 Expression on the Pax2.1 (Noi) Function. Development.

[36] Majumdar A., Lun K., Brand M., Drummond I.A. (2000). Zebrafish No Isthmus Reveals a Role for Pax2.1 in Tubule Differentiation and Patterning Events in the Pronephric Primordia. Development.

[37] Naylor R.W., Skvarca L.B., Thisse C., Thisse B., Hukriede N.A., Davidson A.J. (2016). BMP and Retinoic Acid Regulate Anterior–Posterior Patterning of the Non-Axial Mesoderm across the Dorsal–Ventral Axis. Nat. Commun..

[38] Wingert R.A., Davidson A.J. (2011). Zebrafish Nephrogenesis Involves Dynamic Spatiotemporal Expression Changes in Renal Progenitors and Essential Signals from Retinoic Acid and Irx3b. Dev. Dyn..

[39] James R.G., Kamei C.N., Wang Q., Jiang R., Schultheiss T.M. (2006). Odd-Skipped Related 1 Is Required for Development of the Metanephric Kidney and Regulates Formation and Differentiation of Kidney Precursor Cells. Development.

[40] Mugford J.W., Sipilä P., McMahon J.A., McMahon A.P. (2008). Osr1 Expression Demarcates a Multi-Potent Population of Intermediate Mesoderm That Undergoes Progressive Restriction to an Osr1-Dependent Nephron Progenitor Compartment within the Mammalian Kidney. Dev. Biol..

[41] Zhang X., Lin Q., Ren F., Zhang J., Dawar F.U., Mei J. (2018). The Dysregulated Autophagy Signaling Is Partially Responsible for Defective Podocyte Development in *Wt1a* Mutant Zebrafish. Aquac. Fish..

[42] Elmonem M.A., Berlingerio S.P., Van den Heuvel L.P., De Witte P.A., Lowe M., Levtchenko E.N. (2018). Genetic Renal Diseases: The Emerging Role of Zebrafish Models. Cells.

[43] Grote D., Boualia S.K., Souabni A., Merkel C., Chi X., Costantini F., Carroll T., Bouchard M. (2008). Gata3 Acts Downstream of β-Catenin Signaling to Prevent Ectopic Metanephric Kidney Induction. PLoS Genet..

[44] Outtandy P., Russell C., Kleta R., Bockenhauer D. (2019). Zebrafish as a Model for Kidney Function and Disease. Pediatr. Nephrol..

[45] Carroll T.J., Park J.-S., Hayashi S., Majumdar A., McMahon A.P. (2005). Wnt9b Plays a Central Role in the Regulation of Mesenchymal to Epithelial Transitions Underlying Organogenesis of the Mammalian Urogenital System. Dev. Cell.

[46] Naylor R.W., Przepiorski A., Ren Q., Yu J., Davidson A.J. (2012). HNF1β Is Essential for Nephron Segmentation during Nephrogenesis. J. Am. Soc. Nephrol..

[47] Massa F., Garbay S., Bouvier R., Sugitani Y., Noda T., Gubler M.-C., Heidet L., Pontoglio M., Fischer E. (2013). Hepatocyte Nuclear Factor 1β Controls Nephron Tubular Development. Development.

[48] Drummond I. (2003). Making a Zebrafish Kidney: A Tale of Two Tubes. Trends Cell Biol..

[49] McMahon A.P. (2016). Development of the Mammalian Kidney. Curr. Top. Dev. Biol..

[50] Perner B., Englert C., Bollig F. (2007). The Wilms Tumor Genes *Wt1a* and *Wt1b* Control Different Steps during Formation of the Zebrafish Pronephros. Dev. Biol..

[51] Anzenberger U., Bit-Avragim N., Rohr S., Rudolph F., Dehmel B., Willnow T.E., Abdelilah-Seyfried S. (2006). Elucidation of Megalin/LRP2-Dependent Endocytic Transport Processes in the Larval Zebrafish Pronephros. J. Cell Sci..

[52] Jain S., Chen F. (2018). Developmental Pathology of Congenital Kidney and Urinary Tract Anomalies. Clin. Kidney J..

[53] De Tomasi L., David P., Humbert C., Silbermann F., Arrondel C., Tores F., Fouquet S., Desgrange A., Niel O., Bole-Feysot C. (2017). Mutations in GREB1L Cause Bilateral Kidney Agenesis in Humans and Mice. Am. J. Hum. Genet..

[54] Lee K.H., Gee H.Y., Shin J.I. (2017). Genetics of Vesicoureteral Reflux and Congenital Anomalies of the Kidney and Urinary Tract. Investig. Clin. Urol..

[55] Zhao E., Bomback M., Khan A., Murthy S.K., Solowiejczyk D., Vora N.L., Gilmore K.L., Giordano J.L., Wapner R.J., Sanna-Cherchi S. (2024). The Expanded Spectrum of Human Disease Associated with GREB1L Likely Includes Complex Congenital Heart Disease. Prenat. Diagn..

[56] Al-Hamed M.H., Altuwaijri N., Alsahan N., Ali W., Abdulwahab F., Alzahrani F., Majrashi N., Alkuraya F.S. (2022). A Null Founder Variant in NPNT, Encoding Nephronectin, Causes Autosomal Recessive Renal Agenesis. Clin. Genet..

[57] Kolvenbach C.M., Dworschak G.C., Frese S., Japp A.S., Schuster P., Wenzlitschke N., Yilmaz Ö., Lopes F.M., Pryalukhin A., Schierbaum L. (2019). Rare Variants in BNC2 Are Implicated in Autosomal-Dominant Congenital Lower Urinary-Tract Obstruction. Am. J. Hum. Genet..

[58] Fernandez-Prado R., Kanbay M., Ortiz A., Perez-Gomez M.V. (2019). Expanding Congenital Abnormalities of the Kidney and Urinary Tract (CAKUT) Genetics: Basonuclin 2 (BNC2) and Lower Urinary Tract Obstruction. Ann. Transl. Med..

[59] Sanna-Cherchi S., Sampogna R.V., Papeta N., Burgess K.E., Nees S.N., Perry B.J., Choi M., Bodria M., Liu Y., Weng P.L. (2013). Mutations in DSTYK and Dominant Urinary Tract Malformations. N. Engl. J. Med..

[60] Bower M.A., Schimmenti L.A., Eccles M.R., Adam M.P., Bick S., Mirzaa G.M., Pagon R.A., Wallace S.E., Amemiya A. (1993). PAX2-Related Disorder. GeneReviews^®^.

[61] Vivante A., Mark-Danieli M., Davidovits M., Harari-Steinberg O., Omer D., Gnatek Y., Cleper R., Landau D., Kovalski Y., Weissman I. (2013). Renal Hypodysplasia Associates with a Wnt4 Variant That Causes Aberrant Canonical Wnt Signaling. J. Am. Soc. Nephrol..

[62] Weber S., Taylor J.C., Winyard P., Baker K.F., Sullivan-Brown J., Schild R., Knüppel T., Zurowska A.M., Caldas-Alfonso A., Litwin M. (2008). SIX2 and BMP4 Mutations Associate with Anomalous Kidney Development. J. Am. Soc. Nephrol..

[63] Faguer S., Chassaing N., Bandin F., Prouheze C., Chauveau D., Decramer S. (2012). Should *SIX2* Be Routinely Tested in Patients with Isolated Congenital Abnormalities of Kidneys and/or Urinary Tract (CAKUT)?. Eur. J. Med. Genet..

[64] Saburi S., Hester I., Fischer E., Pontoglio M., Eremina V., Gessler M., Quaggin S.E., Harrison R., Mount R., McNeill H. (2008). Loss of Fat4 Disrupts PCP Signaling and Oriented Cell Division and Leads to Cystic Kidney Disease. Nat. Genet..

[65] Naylor R.W., Chang H.-H.G., Qubisi S., Davidson A.J. (2018). A Novel Mechanism of Gland Formation in Zebrafish Involving Transdifferentiation of Renal Epithelial Cells and Live Cell Extrusion. eLife.

[66] Sun Z., Hopkins N. (2001). Vhnf1, the MODY5 and Familial GCKD-Associated Gene, Regulates Regional Specification of the Zebrafish Gut, Pronephros, and Hindbrain. Genes Dev..

[67] Sheehan-Rooney K., Swartz M.E., Zhao F., Liu D., Eberhart J.K. (2013). Ahsa1 and Hsp90 Activity Confers More Severe Craniofacial Phenotypes in a Zebrafish Model of Hypoparathyroidism, Sensorineural Deafness and Renal Dysplasia (HDR). Dis. Model. Mech..

[68] Belcher B., Vestal J., Lane S., Kell M., Smith L., Camarata T. (2023). The Zebrafish Paralog Six2b Is Required for Early Proximal Pronephros Morphogenesis. Sci. Rep..

[69] Kroeger P.T., Drummond B.E., Miceli R., McKernan M., Gerlach G.F., Marra A.N., Fox A., McCampbell K.K., Leshchiner I., Rodriguez-Mari A. (2017). The Zebrafish Kidney Mutant *Zeppelin* Reveals That *Brca2/Fancd1* Is Essential for Pronephros Development. Dev. Biol..

[70] Gerlach G.F., Wingert R.A. (2014). Zebrafish Pronephros Tubulogenesis and Epithelial Identity Maintenance Are Reliant on the Polarity Proteins Prkc Iota and Zeta. Dev. Biol..

[71] Stark K., Vainio S., Vassileva G., McMahon A.P. (1994). Epithelial Transformation of Metanephric Mesenchyme in the Developing Kidney Regulated by Wnt-4. Nature.

[72] Kispert A., Vainio S., McMahon A.P. (1998). Wnt-4 Is a Mesenchymal Signal for Epithelial Transformation of Metanephric Mesenchyme in the Developing Kidney. Development.

[73] Saulnier D.M.E., Ghanbari H., Brändli A.W. (2002). Essential Function of Wnt-4 for Tubulogenesis in the *Xenopus* Pronephric Kidney. Dev. Biol..

[74] Tanigawa S., Wang H., Yang Y., Sharma N., Tarasova N., Ajima R., Yamaguchi T.P., Rodriguez L.G., Perantoni A.O. (2011). Wnt4 Induces Nephronic Tubules in Metanephric Mesenchyme by a Non-Canonical Mechanism. Dev. Biol..

[75] Lyons J.P., Miller R.K., Zhou X., Weidinger G., Deroo T., Denayer T., Park J., Ji H., Hong J.Y., Li A. (2009). Requirement of Wnt/β-catenin signaling in pronephric kidney development. Mech. Dev..

[76] Lopez-Rivera E., Liu Y.P., Verbitsky M., Anderson B.R., Capone V.P., Otto E.A., Yan Z., Mitrotti A., Martino J., Steers N.J. (2017). Genetic Drivers of Kidney Defects in the DiGeorge Syndrome. N. Engl. J. Med..

[77] Moon A.M., Guris D.L., Seo J., Li L., Hammond J., Talbot A., Imamoto A. (2006). Crkl Deficiency Disrupts Fgf8 Signaling in a Mouse Model of 22q11 Deletion Syndromes. Dev. Cell.

[78] Du J., Meng L., Pang L., Jin B., Duan N., Huang C., Huang H., Li H. (2020). Crk1/2 and CrkL Play Critical Roles in Maintaining Podocyte Morphology and Function. Exp. Cell Res..

[79] Skouloudaki K., Puetz M., Simons M., Courbard J.-R., Boehlke C., Hartleben B., Engel C., Moeller M.J., Englert C., Bollig F. (2009). Scribble Participates in Hippo Signaling and Is Required for Normal Zebrafish Pronephros Development. Proc. Natl. Acad. Sci. USA.

[80] Walma D.A.C., Yamada K.M. (2020). The Extracellular Matrix in Development. Development.

[81] Rozario T., DeSimone D.W. (2010). The Extracellular Matrix in Development and Morphogenesis: A Dynamic View. Dev. Biol..

[82] Müller-Deile J., Sopel N., Ohs A., Rose V., Gröner M., Wrede C., Hegermann J., Daniel C., Amann K., Zahner G. (2021). Glomerular Endothelial Cell-Derived microRNA-192 Regulates Nephronectin Expression in Idiopathic Membranous Glomerulonephritis. J. Am. Soc. Nephrol..

[83] Patra C., Rayrikar A., Wagh G., Kleefeldt F., Roshanbinfar K., Cop F., Nikolic I., Schmidt M.H.H., Acker-Palmer A., Ergün S. (2025). Nephronectin Is Required for Vascularization in Zebrafish and Sufficient to Promote Mammalian Vessel-Like Structures in Hydrogels for Tissue Engineering. J. Am. Heart Assoc..

[84] Carney T.J., Feitosa N.M., Sonntag C., Slanchev K., Kluger J., Kiyozumi D., Gebauer J.M., Talbot J.C., Kimmel C.B., Sekiguchi K. (2010). Genetic Analysis of Fin Development in Zebrafish Identifies Furin and Hemicentin1 as Potential Novel Fraser Syndrome Disease Genes. PLoS Genet..

[85] Naylor R.W., Lemarie E., Jackson-Crawford A., Davenport J.B., Mironov A., Lowe M., Lennon R. (2022). A Novel Nanoluciferase Transgenic Reporter Measures Proteinuria in Zebrafish. Kidney Int..

[86] LeBleu V.S., Dai J., Tsutakawa S., MacDonald B.A., Alge J.L., Sund M., Xie L., Sugimoto H., Tainer J., Zon L.I. (2023). Identification of Unique A4 Chain Structure and Conserved Antiangiogenic Activity of α3NC1 Type IV Collagen in Zebrafish. Dev. Dyn..

[87] Dai L., Li J., Xie L., Wang W., Lu Y., Xie M., Huang J., Shen K., Yang H., Pei C. (2021). A Biallelic Frameshift Mutation in Nephronectin Causes Bilateral Renal Agenesis in Humans. J. Am. Soc. Nephrol..

[88] Bill B.R., Petzold A.M., Clark K.J., Schimmenti L.A., Ekker S.C. (2009). A Primer for Morpholino Use in Zebrafish. Zebrafish.

[89] Bedell V.M., Westcot S.E., Ekker S.C. (2011). Lessons from Morpholino-Based Screening in Zebrafish. Brief. Funct. Genom..

[90] Stainier D.Y.R., Raz E., Lawson N.D., Ekker S.C., Burdine R.D., Eisen J.S., Ingham P.W., Schulte-Merker S., Yelon D., Weinstein B.M. (2017). Guidelines for Morpholino Use in Zebrafish. PLoS Genet..

[91] Hwang W.Y., Fu Y., Reyon D., Maeder M.L., Tsai S.Q., Sander J.D., Peterson R.T., Yeh J.-R.J., Joung J.K. (2013). Efficient In Vivo Genome Editing Using RNA-Guided Nucleases. Nat. Biotechnol..

[92] Li M., Zhao L., Page-McCaw P., Chen W. (2016). Zebrafish Genome Engineering Using the CRISPR-Cas9 System. Trends Genet..

[93] Kroll F., Powell G.T., Ghosh M., Gestri G., Antinucci P., Hearn T.J., Tunbak H., Lim S., Dennis H.W., Fernandez J.M. (2021). A Simple and Effective F0 Knockout Method for Rapid Screening of Behaviour and Other Complex Phenotypes. eLife.

[94] Medishetti R., Balamurugan K., Yadavalli K., Rani R., Sevilimedu A., Challa A.K., Parsa K., Chatti K. (2022). CRISPR-Cas9-Induced Gene Knockout in Zebrafish. STAR Protoc..

[95] Quick R.E., Buck L.D., Parab S., Tolbert Z.R., Matsuoka R.L. (2021). Highly Efficient Synthetic CRISPR RNA/Cas9-Based Mutagenesis for Rapid Cardiovascular Phenotypic Screening in F0 Zebrafish. Front. Cell Dev. Biol..

[96] Hoshijima K., Jurynec M.J., Shaw D.K., Jacobi A.M., Behlke M.A., Grunwald D.J. (2019). Highly Efficient Methods for Generating Deletion Mutations and F0 Embryos That Lack Gene Function in Zebrafish. Dev. Cell.

[97] Rossi A., Kontarakis Z., Gerri C., Nolte H., Hölper S., Krüger M., Stainier D.Y.R. (2015). Genetic Compensation Induced by Deleterious Mutations but Not Gene Knockdowns. Nature.

[98] Kok F.O., Shin M., Ni C.-W., Gupta A., Grosse A.S., van Impel A., Kirchmaier B.C., Peterson-Maduro J., Kourkoulis G., Male I. (2015). Reverse Genetic Screening Reveals Poor Correlation between Morpholino-Induced and Mutant Phenotypes in Zebrafish. Dev. Cell.

[99] Flannery K.P., Mowla S., Battula N., Clark L.R., Oliveira C.D., Simhon L.M., Liu D., Venkatesan C., Karas B.F., Terez K.R. (2026). Impact of Maternal Compensation on Developmental Phenotypes in a Zebrafish Model of Severe Congenital Muscular Dystrophy. PLoS Genet..

[100] Robu M.E., Larson J.D., Nasevicius A., Beiraghi S., Brenner C., Farber S.A., Ekker S.C. (2007). P53 Activation by Knockdown Technologies. PLoS Genet..

[101] Eisen J.S., Smith J.C. (2008). Controlling Morpholino Experiments: Don’t Stop Making Antisense. Development.

[102] Hruscha A., Krawitz P., Rechenberg A., Heinrich V., Hecht J., Haass C., Schmid B. (2013). Efficient CRISPR/Cas9 Genome Editing with Low off-Target Effects in Zebrafish. Development.

[103] Auer T.O., Duroure K., De Cian A., Concordet J.-P., Del Bene F. (2014). Highly Efficient CRISPR/Cas9-Mediated Knock-in in Zebrafish by Homology-Independent DNA Repair. Genome Res..

[104] Vanhooydonck M., De Neef E., De Saffel H., Boel A., Willaert A., Callewaert B., Claes K.B.M. (2025). Prime Editing Outperforms Homology-Directed Repair as a Tool for CRISPR-Mediated Variant Knock-in in Zebrafish. Lab Anim..

[105] de Vrieze E., de Bruijn S.E., Reurink J., Broekman S., van de Riet V., Aben M., Kremer H., van Wijk E. (2021). Efficient Generation of Knock-In Zebrafish Models for Inherited Disorders Using CRISPR-Cas9 Ribonucleoprotein Complexes. Int. J. Mol. Sci..

[106] Albadri S., Del Bene F., Revenu C. (2017). Genome Editing Using CRISPR/Cas9-Based Knock-in Approaches in Zebrafish. Methods.

[107] Prykhozhij S.V., Fuller C., Steele S.L., Veinotte C.J., Razaghi B., Robitaille J.M., McMaster C.R., Shlien A., Malkin D., Berman J.N. (2018). Optimized Knock-in of Point Mutations in Zebrafish Using CRISPR/Cas9. Nucleic Acids Res..

[108] Carrington B., Ramanagoudr-Bhojappa R., Bresciani E., Han T.-U., Sood R. (2022). A Robust Pipeline for Efficient Knock-in of Point Mutations and Epitope Tags in Zebrafish Using Fluorescent PCR Based Screening. BMC Genom..

[109] Qi L.S., Larson M.H., Gilbert L.A., Doudna J.A., Weissman J.S., Arkin A.P., Lim W.A. (2013). Repurposing CRISPR as an RNA-Guided Platform for Sequence-Specific Control of Gene Expression. Cell.

[110] Larson M.H., Gilbert L.A., Wang X., Lim W.A., Weissman J.S., Qi L.S. (2013). CRISPR Interference (CRISPRi) for Sequence-Specific Control of Gene Expression. Nat. Protoc..

[111] Chan J., Wu Z., Liu M., Wang T., Liu H., Cao R., Li X., Li X., Zhan S., Cheng J. (2025). Systematic Enhancer Mapping and Functional Analysis in Zebrafish with Optimized CRISPR Interference. Nucleic Acids Res..

[112] Cornet C., Di Donato V., Terriente J. (2018). Combining Zebrafish and CRISPR/Cas9: Toward a More Efficient Drug Discovery Pipeline. Front. Pharmacol..

[113] Gilbert L.A., Larson M.H., Morsut L., Liu Z., Brar G.A., Torres S.E., Stern-Ginossar N., Brandman O., Whitehead E.H., Doudna J.A. (2013). CRISPR-Mediated Modular RNA-Guided Regulation of Transcription in Eukaryotes. Cell.

[114] Konermann S., Brigham M.D., Trevino A.E., Joung J., Abudayyeh O.O., Barcena C., Hsu P.D., Habib N., Gootenberg J.S., Nishimasu H. (2015). Genome-Scale Transcriptional Activation by an Engineered CRISPR-Cas9 Complex. Nature.

[115] Perez-Pinera P., Kocak D.D., Vockley C.M., Adler A.F., Kabadi A.M., Polstein L.R., Thakore P.I., Glass K.A., Ousterout D.G., Leong K.W. (2013). RNA-Guided Gene Activation by CRISPR-Cas9-Based Transcription Factors. Nat. Methods.

[116] Maeder M.L., Linder S.J., Cascio V.M., Fu Y., Ho Q.H., Joung J.K. (2013). CRISPR RNA-Guided Activation of Endogenous Human Genes. Nat. Methods.

[117] Long L., Guo H., Yao D., Xiong K., Li Y., Liu P., Zhu Z., Liu D. (2015). Regulation of Transcriptionally Active Genes via the Catalytically Inactive Cas9 in *C. elegans* and *D. rerio*. Cell Res..

[118] Barrientos N.B., Shoppell E.A., Boyd R.J., Culotta V.C., McCallion A.S. (2025). Proof of Concept Study Deploying CRISPR Inhibition and Activation Opens Key Avenues for Systematic Biological Exploration in Zebrafish. bioRxiv.

[119] Shi M., Ge W., Li C., Liu B., Deng X., Liu C., Zheng M., Zhang P., Li L., Guo Y. (2026). Versatile CRISPR-Cas Tools for Gene Regulation in Zebrafish via an Enhanced Q Binary System. Adv. Sci..

[120] Westland R., Verbitsky M., Vukojevic K., Perry B.J., Fasel D.A., Zwijnenburg P.J.G., Bökenkamp A., Gille J.J.P., Saraga-Babic M., Ghiggeri G.M. (2015). Copy Number Variation Analysis Identifies Novel CAKUT Candidate Genes in Children with a Solitary Functioning Kidney. Kidney Int..

[121] He B., Ebarasi L., Hultenby K., Tryggvason K., Betsholtz C. (2011). Podocin-Green Fluorescence Protein Allows Visualization and Functional Analysis of Podocytes. J. Am. Soc. Nephrol..

[122] Horsfield J., Ramachandran A., Reuter K., LaVallie E., Collins-Racie L., Crosier K., Crosier P. (2002). Cadherin-17 Is Required to Maintain Pronephric Duct Integrity during Zebrafish Development. Mech. Dev..

[123] Sanker S., Cirio M.C., Vollmer L.L., Goldberg N.D., McDermott L.A., Hukriede N.A., Vogt A. (2013). Development of High-Content Assays for Kidney Progenitor Cell Expansion in Transgenic Zebrafish. J. Biomol. Screen..

[124] Thisse C., Thisse B. (2008). High-Resolution in Situ Hybridization to Whole-Mount Zebrafish Embryos. Nat. Protoc..

[125] Staudt N., Müller-Sienerth N., Fane-Dremucheva A., Yusaf S.P., Millrine D., Wright G.J. (2015). A Panel of Recombinant Monoclonal Antibodies against Zebrafish Neural Receptors and Secreted Proteins Suitable for Wholemount Immunostaining. Biochem. Biophys. Res. Commun..

[126] Deflorian G., Cinquanta M., Beretta C., Venuto A., Santoriello C., Baldessari D., Pezzimenti F., Aliprandi M., Mione M., de Marco A. (2009). Monoclonal Antibodies Isolated by Large-Scale Screening Are Suitable for Labeling Adult Zebrafish (Danio Rerio) Tissues and Cell Structures. J. Immunol. Methods.

[127] Ichimura K., Fukuyo Y., Nakamura T., Powell R., Sakai T., Janknecht R., Obara T. (2013). Developmental Localization of Nephrin in Zebrafish and Medaka Pronephric Glomerulus. J. Histochem. Cytochem..

[128] Fukuyo Y., Nakamura T., Bubenshchikova E., Powell R., Tsuji T., Janknecht R., Obara T. (2014). Nephrin and Podocin Functions Are Highly Conserved between the Zebrafish Pronephros and Mammalian Metanephros. Mol. Med. Rep..

[129] Finckbeiner S., Ko P.-J., Carrington B., Sood R., Gross K., Dolnick B., Sufrin J., Liu P. (2011). Transient Knockdown and Overexpression Reveal a Developmental Role for the Zebrafish *enosf1b* Gene. Cell Biosci..

[130] Sorrells S., Toruno C., Stewart R.A., Jette C. (2013). Analysis of Apoptosis in Zebrafish Embryos by Whole-Mount Immunofluorescence to Detect Activated Caspase 3. J. Vis. Exp..

[131] Sanna-Cherchi S., Khan K., Westland R., Krithivasan P., Fievet L., Rasouly H.M., Ionita-Laza I., Capone V.P., Fasel D.A., Kiryluk K. (2017). Exome-Wide Association Study Identifies GREB1L Mutations in Congenital Kidney Malformations. Am. J. Hum. Genet..

[132] Harnish J.M., Deal S.L., Chao H.-T., Wangler M.F., Yamamoto S. (2019). In Vivo Functional Study of Disease-Associated Rare Human Variants Using Drosophila. J. Vis. Exp..

[133] Kostiuk V., Khokha M.K. (2021). *Xenopus* as a Platform for Discovery of Genes Relevant to Human Disease. Curr. Top. Dev. Biol..

[134] Ecovoiu A.A., Ratiu A.C., Micheu M.M., Chifiriuc M.C. (2022). Inter-Species Rescue of Mutant Phenotype—The Standard for Genetic Analysis of Human Genetic Disorders in Drosophila Melanogaster Model. Int. J. Mol. Sci..

[135] Hartill V.L., van de Hoek G., Patel M.P., Little R., Watson C.M., Berry I.R., Shoemark A., Abdelmottaleb D., Parkes E., Bacchelli C. (2018). DNAAF1 Links Heart Laterality with the AAA+ ATPase RUVBL1 and Ciliary Intraflagellar Transport. Hum. Mol. Genet..

[136] Loontiens S., Depestel L., Vanhauwaert S., Dewyn G., Gistelinck C., Verboom K., Van Loocke W., Matthijssens F., Willaert A., Vandesompele J. (2019). Purification of High-Quality RNA from a Small Number of Fluorescence Activated Cell Sorted Zebrafish Cells for RNA Sequencing Purposes. BMC Genom..

[137] Lawson N.D., Li R., Shin M., Grosse A., Yukselen O., Stone O.A., Kucukural A., Zhu L. (2020). An Improved Zebrafish Transcriptome Annotation for Sensitive and Comprehensive Detection of Cell Type-Specific Genes. Elife.

[138] Grand K., Stoltz M., Rizzo L., Röck R., Kaminski M.M., Salinas G., Getwan M., Naert T., Pichler R., Lienkamp S.S. (2023). HNF1B Alters an Evolutionarily Conserved Nephrogenic Program of Target Genes. J. Am. Soc. Nephrol..

[139] Harder J.L., Menon R., Otto E.A., Zhou J., Eddy S., Wys N.L., O’Connor C., Luo J., Nair V., Cebrian C. (2019). Organoid Single Cell Profiling Identifies a Transcriptional Signature of Glomerular Disease. JCI Insight.

[140] Wu H., Uchimura K., Donnelly E., Kirita Y., Morris S.A., Humphreys B.D. (2018). Comparative Analysis and Refinement of Human PSC-Derived Kidney Organoid Differentiation with Single Cell Transcriptomics. Cell Stem Cell.

[141] Chia I., Grote D., Marcotte M., Batourina E., Mendelsohn C., Bouchard M. (2011). Nephric Duct Insertion Is a Crucial Step in Urinary Tract Maturation That Is Regulated by a Gata3-Raldh2-Ret Molecular Network in Mice. Development.

[142] Belge H., Dahan K., Cambier J.-F., Benoit V., Morelle J., Bloch J., Vanhille P., Pirson Y., Demoulin N. (2017). Clinical and Mutational Spectrum of Hypoparathyroidism, Deafness and Renal Dysplasia Syndrome. Nephrol. Dial. Transplant..

[143] Silva E., Tsatskis Y., Gardano L., Tapon N., McNeill H. (2006). The Tumor-Suppressor Gene Fat Controls Tissue Growth Upstream of Expanded in the Hippo Signaling Pathway. Curr. Biol..

[144] Willecke M., Hamaratoglu F., Kango-Singh M., Udan R., Chen C., Tao C., Zhang X., Halder G. (2006). The Fat Cadherin Acts through the Hippo Tumor-Suppressor Pathway to Regulate Tissue Size. Curr. Biol..

[145] Lelongt B., Ronco P. (2003). Role of Extracellular Matrix in Kidney Development and Repair. Pediatr. Nephrol..

[146] Jessen J.R. (2015). Recent Advances in the Study of Zebrafish Extracellular Matrix Proteins. Dev. Biol..

[147] Linton J.M., Martin G.R., Reichardt L.F. (2007). The ECM Protein Nephronectin Promotes Kidney Development via Integrin A8β1-Mediated Stimulation of Gdnf Expression. Development.

[148] Richards S., Aziz N., Bale S., Bick D., Das S., Gastier-Foster J., Grody W.W., Hegde M., Lyon E., Spector E. (2015). Standards and Guidelines for the Interpretation of Sequence Variants: A Joint Consensus Recommendation of the American College of Medical Genetics and Genomics and the Association for Molecular Pathology. Genet. Med..

[149] Takasato M., Er P.X., Chiu H.S., Maier B., Baillie G.J., Ferguson C., Parton R.G., Wolvetang E.J., Roost M.S., Chuva de Sousa Lopes S.M. (2015). Kidney Organoids from Human iPS Cells Contain Multiple Lineages and Model Human Nephrogenesis. Nature.

[150] Freedman B.S., Brooks C.R., Lam A.Q., Fu H., Morizane R., Agrawal V., Saad A.F., Li M.K., Hughes M.R., Werff R.V. (2015). Modelling Kidney Disease with CRISPR-Mutant Kidney Organoids Derived from Human Pluripotent Epiblast Spheroids. Nat. Commun..

[151] Ungricht R., Guibbal L., Lasbennes M.-C., Orsini V., Beibel M., Waldt A., Cuttat R., Carbone W., Basler A., Roma G. (2022). Genome-Wide Screening in Human Kidney Organoids Identifies Developmental and Disease-Related Aspects of Nephrogenesis. Cell Stem Cell.

[152] Homan K.A., Gupta N., Kroll K.T., Kolesky D.B., Skylar-Scott M., Miyoshi T., Mau D., Valerius M.T., Ferrante T., Bonventre J.V. (2019). Flow-Enhanced Vascularization and Maturation of Kidney Organoids in Vitro. Nat. Methods.

[153] Subramanian A., Sidhom E.-H., Emani M., Vernon K., Sahakian N., Zhou Y., Kost-Alimova M., Slyper M., Waldman J., Dionne D. (2019). Single Cell Census of Human Kidney Organoids Shows Reproducibility and Diminished Off-Target Cells after Transplantation. Nat. Commun..

[154] Lindström N.O., De Sena Brandine G., Tran T., Ransick A., Suh G., Guo J., Kim A.D., Parvez R.K., Ruffins S.W., Rutledge E.A. (2018). Progressive Recruitment of Mesenchymal Progenitors Reveals a Time-Dependent Process of Cell Fate Acquisition in Mouse and Human Nephrogenesis. Dev. Cell.

[155] Mi H., Muruganujan A., Huang X., Ebert D., Mills C., Guo X., Thomas P.D. (2019). Protocol Update for Large-Scale Genome and Gene Function Analysis with the PANTHER Classification System (v.14.0). Nat. Protoc..

[156] Wei M., Yu Q., Li E., Zhao Y., Sun C., Li H., Liu Z., Ji G. (2024). Ace Deficiency Induces Intestinal Inflammation in Zebrafish. Int. J. Mol. Sci..

[157] Esther C.R., Howard T.E., Marino E.M., Goddard J.M., Capecchi M.R., Bernstein K.E. (1996). Mice Lacking Angiotensin-Converting Enzyme Have Low Blood Pressure, Renal Pathology, and Reduced Male Fertility. Lab. Investig..

[158] Kim G.J., Melgoza A., Jiang F., Guo S. (2021). The Effect of Renin–Angiotensin–Aldosterone System Inhibitors on Organ-Specific Ace2 Expression in Zebrafish and Its Implications for COVID-19. Sci. Rep..

[159] Kim H.S., Krege J.H., Kluckman K.D., Hagaman J.R., Hodgin J.B., Best C.F., Jennette J.C., Coffman T.M., Maeda N., Smithies O. (1995). Genetic Control of Blood Pressure and the Angiotensinogen Locus. Proc. Natl. Acad. Sci. USA.

[160] Oliverio M.I., Kim H.S., Ito M., Le T., Audoly L., Best C.F., Hiller S., Kluckman K., Maeda N., Smithies O. (1998). Reduced Growth, Abnormal Kidney Structure, and Type 2 (AT2) Angiotensin Receptor-Mediated Blood Pressure Regulation in Mice Lacking Both AT1A and AT1B Receptors for Angiotensin II. Proc. Natl. Acad. Sci. USA.

[161] Klämbt V., Buerger F., Wang C., Naert T., Richter K., Nauth T., Weiss A.-C., Sieckmann T., Lai E., Connaughton D.M. (2023). Genetic Variants in *ARHGEF6* Cause Congenital Anomalies of the Kidneys and Urinary Tract in Humans, Mice, and Frogs. J. Am. Soc. Nephrol..

[162] Lang M.R., Patterson L.B., Gordon T.N., Johnson S.L., Parichy D.M. (2009). Basonuclin-2 Requirements for Zebrafish Adult Pigment Pattern Development and Female Fertility. PLoS Genet..

[163] Burczyk M.S., Burkhalter M.D., Tena T.C., Grisanti L.A., Kauk M., Matysik S., Donow C., Kustermann M., Rothe M., Cui Y. (2019). Muscarinic Receptors Promote Pacemaker Fate at the Expense of Secondary Conduction System Tissue in Zebrafish. JCI Insight.

[164] Matsui M., Motomura D., Karasawa H., Fujikawa T., Jiang J., Komiya Y., Takahashi S., Taketo M.M. (2000). Multiple Functional Defects in Peripheral Autonomic Organs in Mice Lacking Muscarinic Acetylcholine Receptor Gene for the M3 Subtype. Proc. Natl. Acad. Sci. USA.

[165] Rima M., Lattouf Y., Abi Younes M., Bullier E., Legendre P., Mangin J.-M., Hong E. (2020). Dynamic Regulation of the Cholinergic System in the Spinal Central Nervous System. Sci. Rep..

[166] Raine J., Kibat C., Banerjee T.D., Monteiro A., Mathuru A.S. (2025). *chrna3* Modulates Alcohol Response. J. Neurosci..

[167] Pöschl E., Schlötzer-Schrehardt U., Brachvogel B., Saito K., Ninomiya Y., Mayer U. (2004). Collagen IV Is Essential for Basement Membrane Stability but Dispensable for Initiation of Its Assembly during Early Development. Development.

[168] Chen Z., Migeon T., Verpont M.-C., Zaidan M., Sado Y., Kerjaschki D., Ronco P., Plaisier E. (2016). HANAC Syndrome *Col4a1* Mutation Causes Neonate Glomerular Hyperpermeability and Adult Glomerulocystic Kidney Disease. J. Am. Soc. Nephrol..

[169] Martino J., Liu Q., Vukojevic K., Ke J., Lim T.Y., Khan A., Gupta Y., Perez A., Yan Z., Rasouly H.M. (2023). Mouse and Human Studies Support *DSTYK* Loss of Function as a Low-Penetrance and Variable Expressivity Risk Factor for Congenital Urinary Tract Anomalies. Genet. Med..

[170] Paradisi G., Bonavolontà V., Venditti M., Fasano G., Pedalino C., Del Bene F., Tartaglia M., Lauri A. (2026). Zebrafish *Col4a1* Loss-of-Function Models Mirror Key Neurovascular and Ocular Features of COL4A1/A2 Syndrome and Enable Human Variants Assessment in Vivo. Matrix Biol..

[171] Flatman D., Naylor R.W., Crilly S., Carter I., Mironov A., Pinteaux E., Allan S.M., Lennon R., Kasher P.R. (2025). Loss of *Col4a1* in Zebrafish Recapitulates the Cerebrovascular Phenotypes Associated with Monogenic Cerebral Small Vessel Disease. Matrix Biol..

[172] Stergas H.R., Kalbag Z., St. Clair R.M., Talbot J.C., Ballif B.A., Ebert A.M. (2022). Crk Adaptor Proteins Are Necessary for the Development of the Zebrafish Retina. Dev. Dyn..

[173] Lu B.C., Cebrian C., Chi X., Kuure S., Kuo R., Bates C.M., Arber S., Hassell J., MacNeil L., Hoshi M. (2009). Etv4 and Etv5 Are Required Downstream of GDNF and Ret for Kidney Branching Morphogenesis. Nat. Genet..

[174] Kuure S., Chi X., Lu B., Costantini F. (2010). The Transcription Factors Etv4 and Etv5 Mediate Formation of the Ureteric Bud Tip Domain during Kidney Development. Development.

[175] Marra A.N., Wingert R.A. (2016). Epithelial Cell Fate in the Nephron Tubule Is Mediated by the ETS Transcription Factors *Etv5a* and *Etv4* during Zebrafish Kidney Development. Dev. Biol..

[176] Sahly I., Andermann P., Petit C. (1999). The Zebrafish *Eya1* Gene and Its Expression Pattern during Embryogenesis. Dev. Genes Evol..

[177] Xu P.-X., Adams J., Peters H., Brown M.C., Heaney S., Maas R. (1999). Eya1-Deficient Mice Lack Ears and Kidneys and Show Abnormal Apoptosis of Organ Primordia. Nat. Genet..

[178] Sajithlal G., Zou D., Silvius D., Xu P.-X. (2005). Eya1 Acts as a Critical Regulator for Specifying the Metanephric Mesenchyme. Dev. Biol..

[179] Xu P.-X., Zheng W., Huang L., Maire P., Laclef C., Silvius D. (2003). *Six1* Is Required for the Early Organogenesis of Mammalian Kidney. Development.

[180] Kozlowski D.J., Whitfield T.T., Hukriede N.A., Lam W.K., Weinberg E.S. (2005). The Zebrafish *Dog-Eared* Mutation Disrupts *Eya1*, a Gene Required for Cell Survival and Differentiation in the Inner Ear and Lateral Line. Dev. Biol..

[181] Zhang H., Bagherie-Lachidan M., Badouel C., Enderle L., Peidis P., Bremner R., Kuure S., Jain S., McNeill H. (2019). FAT4 Fine-Tunes Kidney Development by Regulating RET Signaling. Dev. Cell.

[182] Barak H., Huh S.-H., Chen S., Jeanpierre C., Martinovic J., Parisot M., Bole-Feysot C., Nitschké P., Salomon R., Antignac C. (2012). Fgf9 and FGF20 Maintain the Stemness of Nephron Progenitors in Mice and Man. Dev. Cell.

[183] Huh S.-H., Ha L., Jang H.-S. (2020). Nephron Progenitor Maintenance Is Controlled through Fibroblast Growth Factors and Sprouty1 Interaction. J. Am. Soc. Nephrol..

[184] Gallegos T.F., Kamei C.N., Rohly M., Drummond I.A. (2019). Fibroblast Growth Factor Signaling Mediates Progenitor Cell Aggregation and Nephron Regeneration in the Adult Zebrafish Kidney. Dev. Biol..

[185] Cooper W.J., Wirgau R.M., Sweet E.M., Albertson R.C. (2013). Deficiency of Zebrafish *Fgf20a* Results in Aberrant Skull Remodeling That Mimics Both Human Cranial Disease and Evolutionarily Important Fish Skull Morphologies. Evol. Dev..

[186] Whitehead G.G., Makino S., Lien C.-L., Keating M.T. (2005). *fgf20* Is Essential for Initiating Zebrafish Fin Regeneration. Science.

[187] Hawkey-Noble A., Pater J.A., Kollipara R., Fitzgerald M., Maekawa A.S., Kovacs C.S., Young T.-L., French C.R. (2022). Mutation of *foxl1* Results in Reduced Cartilage Markers in a Zebrafish Model of Otosclerosis. Genes.

[188] Kume T., Deng K., Hogan B.L.M. (2000). Murine Forkhead/Winged Helix Genes *Foxc1* (*Mf1*) and *Foxc2* (*Mfh1*) Are Required for the Early Organogenesis of the Kidney and Urinary Tract. Development.

[189] Motojima M., Tanimoto S., Ohtsuka M., Matsusaka T., Kume T., Abe K. (2016). Characterization of Kidney and Skeleton Phenotypes of Mice Double Heterozygous for *Foxc1* and *Foxc2*. Cells Tissues Organs.

[190] O’Brien L.L., Grimaldi M., Kostun Z., Wingert R.A., Selleck R., Davidson A.J. (2011). Wt1a, Foxc1a, and the Notch Mediator Rbpj Physically Interact and Regulate the Formation of Podocytes in Zebrafish. Dev. Biol..

[191] Ferre-Fernández J.-J., Sorokina E.A., Thompson S., Collery R.F., Nordquist E., Lincoln J., Semina E.V. (2020). Disruption of Foxc1 Genes in Zebrafish Results in Dosage-Dependent Phenotypes Overlapping Axenfeld-Rieger Syndrome. Hum. Mol. Genet..

[192] Topczewska J.M., Topczewski J., Shostak A., Kume T., Solnica-Krezel L., Hogan B.L.M. (2001). The Winged Helix Transcription Factor Foxc1a Is Essential for Somitogenesis in Zebrafish. Genes Dev..

[193] Umali J., Hawkey-Noble A., French C.R. (2019). Loss of Foxc1 in Zebrafish Reduces Optic Nerve Size and Cell Number in the Retinal Ganglion Cell Layer. Vis. Res..

[194] White J.T., Zhang B., Cerqueira D.M., Tran U., Wessely O. (2010). Notch Signaling, Wt1 and Foxc2 Are Key Regulators of the Podocyte Gene Regulatory Network in Xenopus. Development.

[195] Cheng L., Chong M., Fan W., Guo X., Zhang W., Yang X., Liu F., Gui Y., Lu D. (2007). Molecular Cloning, Characterization, and Developmental Expression of Foxp1 in Zebrafish. Dev. Genes Evol..

[196] Wu S.-T., Feng Y., Song R., Qi Y., Li L., Lu D., Wang Y., Wu W., Morgan A., Wang X. (2024). Foxp1 Is Required for Renal Intercalated Cell Differentiation and Acid–Base Regulation. J. Am. Soc. Nephrol..

[197] Grundmann S., Lindmayer C., Hans F.P., Hoefer I., Helbing T., Pasterkamp G., Bode C., de Kleijn D., Moser M. (2013). FoxP1 Stimulates Angiogenesis by Repressing the Inhibitory Guidance Protein Semaphorin 5B in Endothelial Cells. PLoS ONE.

[198] Wafer R., Tandon P., Minchin J. (2025). A Quantitative in Vivo CRISPR-Imaging Platform Identifies Regulators of Hyperplastic and Hypertrophic Adipose Morphology in Zebrafish. eLife.

[199] Pitera J.E., Scambler P.J., Woolf A.S. (2008). Fras1, a Basement Membrane-Associated Protein Mutated in Fraser Syndrome, Mediates Both the Initiation of the Mammalian Kidney and the Integrity of Renal Glomeruli. Hum. Mol. Genet..

[200] Talbot J.C., Walker M.B., Carney T.J., Huycke T.R., Yan Y.-L., BreMiller R.A., Gai L., DeLaurier A., Postlethwait J.H., Hammerschmidt M. (2012). *Fras1* Shapes Endodermal Pouch 1 and Stabilizes Zebrafish Pharyngeal Skeletal Development. Development.

[201] Beck T.F., Shchelochkov O.A., Yu Z., Kim B.J., Hernández-García A., Zaveri H.P., Bishop C., Overbeek P.A., Stockton D.W., Justice M.J. (2013). Novel *Frem1*-Related Mouse Phenotypes and Evidence of Genetic Interactions with *Gata4* and *Slit3*. PLoS ONE.

[202] Simikyan R.G., Zhang X., Strelkova O., Li N., Zhu M., Eckhard A., Baranov P.Y., Wu X., Richey L., Indzhykulian A.A. (2025). *Frem2* Knockout Mice Exhibit Fraser Syndrome Phenotypes and Neonatal Lethality Due to Bilateral Renal Agenesis. bioRxiv.

[203] Shepherd I.T., Beattie C.E., Raible D.W. (2001). Functional Analysis of Zebrafish GDNF. Dev. Biol..

[204] Enomoto H., Araki T., Jackman A., Heuckeroth R.O., Snider W.D., Johnson E.M., Milbrandt J. (1998). GFRα1-Deficient Mice Have Deficits in the Enteric Nervous System and Kidneys. Neuron.

[205] Cacalano G., Fariñas I., Wang L.-C., Hagler K., Forgie A., Moore M., Armanini M., Phillips H., Ryan A.M., Reichardt L.F. (1998). GFRα1 Is an Essential Receptor Component for GDNF in the Developing Nervous System and Kidney. Neuron.

[206] Davis T.K., Hoshi M., Jain S. (2013). Stage Specific Requirement of Gfrα1 in the Ureteric Epithelium during Kidney Development. Mech. Dev..

[207] Brophy P.D., Rasmussen M., Parida M., Bonde G., Darbro B.W., Hong X., Clarke J.C., Peterson K.A., Denegre J., Schneider M. (2017). A Gene Implicated in Activation of Retinoic Acid Receptor Targets Is a Novel Renal Agenesis Gene in Humans. Genetics.

[208] Takamiya K., Kostourou V., Adams S., Jadeja S., Chalepakis G., Scambler P.J., Huganir R.L., Adams R.H. (2004). A Direct Functional Link between the Multi-PDZ Domain Protein GRIP1 and the Fraser Syndrome Protein Fras1. Nat. Genet..

[209] Lokmane L., Heliot C., Garcia-Villalba P., Fabre M., Cereghini S. (2010). vHNF1 Functions in Distinct Regulatory Circuits to Control Ureteric Bud Branching and Early Nephrogenesis. Development.

[210] Desgrange A., Heliot C., Skovorodkin I., Akram S.U., Heikkilä J., Ronkainen V.-P., Miinalainen I., Vainio S.J., Cereghini S. (2017). HNF1B Controls Epithelial Organization and Cell Polarity during Ureteric Bud Branching and Collecting Duct Morphogenesis. Development.

[211] Patterson L.T., Pembaur M., Potter S.S. (2001). *Hoxa11* and *Hoxd11* Regulate Branching Morphogenesis of the Ureteric Bud in the Developing Kidney. Development.

[212] Wellik D.M., Hawkes P.J., Capecchi M.R. (2002). *Hox11* Paralogous Genes Are Essential for Metanephric Kidney Induction. Genes Dev..

[213] Guo C., Kaneko S., Sun Y., Huang Y., Vlodavsky I., Li X., Li Z.-R., Li X. (2015). A Mouse Model of Urofacial Syndrome with Dysfunctional Urination. Hum. Mol. Genet..

[214] Roberts N.A., Hilton E.N., Lopes F.M., Singh S., Randles M.J., Gardiner N.J., Chopra K., Coletta R., Bajwa Z., Hall R.J. (2019). *Lrig2* and *Hpse2*, Mutated in Urofacial Syndrome, Pattern Nerves in the Urinary Bladder. Kidney Int..

[215] Müller U., Wang D., Denda S., Meneses J.J., Pedersen R.A., Reichardt L.F. (1997). Integrin A8β1 Is Critically Important for Epithelial–Mesenchymal Interactions during Kidney Morphogenesis. Cell.

[216] Talbot J.C., Nichols J.T., Yan Y.-L., Leonard I.F., BreMiller R.A., Amacher S.L., Postlethwait J.H., Kimmel C.B. (2016). Pharyngeal Morphogenesis Requires Fras1-Itga8-Dependent Epithelial-Mesenchymal Interaction. Dev. Biol..

[217] Pastor-Soler N.M., Sutton T.A., Mang H.E., Kinlough C.L., Gendler S.J., Madsen C.S., Bastacky S.I., Ho J., Al-bataineh M.M., Hallows K.R. (2015). Muc1 Is Protective during Kidney Ischemia-Reperfusion Injury. Am. J. Physiol. Ren. Physiol..

[218] Wesseling J., van der Valk S.W., Vos H.L., Sonnenberg A., Hilkens J. (1995). Episialin (MUC1) Overexpression Inhibits Integrin-Mediated Cell Adhesion to Extracellular Matrix Components. J. Cell Biol..

[219] Houweling A.C., Beaman G.M., Postma A.V., Gainous T.B., Lichtenbelt K.D., Brancati F., Lopes F.M., van der Made I., Polstra A.M., Robinson M.L. (2019). Loss-of-Function Variants in Myocardin Cause Congenital Megabladder in Humans and Mice. J. Clin. Investig..

[220] Vivante A., Mann N., Yonath H., Weiss A.-C., Getwan M., Kaminski M.M., Bohnenpoll T., Teyssier C., Chen J., Shril S. (2017). A Dominant Mutation in Nuclear Receptor Interacting Protein 1 Causes Urinary Tract Malformations via Dysregulation of Retinoic Acid Signaling. J. Am. Soc. Nephrol..

[221] Porteous S., Torban E., Cho N.-P., Cunliffe H., Chua L., McNoe L., Ward T., Souza C., Gus P., Giugliani R. (2000). Primary Renal Hypoplasiain Humans and Mice with *PAX2* Mutations: Evidence of Increased Apoptosis in Fetal Kidneys of *Pax2*^1Neu^ +/– Mutant Mice. Hum. Mol. Genet..

[222] Dziarmaga A., Clark P., Stayner C., Julien J.P., Torban E., Goodyer P., Eccles M. (2003). Ureteric Bud Apoptosis and Renal Hypoplasia in Transgenic *PAX2*-*Bax* Fetal Mice Mimics the Renal-Coloboma Syndrome. J. Am. Soc. Nephrol..

[223] Schnabel C.A., Godin R.E., Cleary M.L. (2003). *Pbx1* Regulates Nephrogenesis and Ureteric Branching in the Developing Kidney. Dev. Biol..

[224] Teoh P.-H., Shu-Chien A.C., Chan W.-K. (2010). *Pbx1* Is Essential for Growth of Zebrafish Swim Bladder. Dev. Dyn..

[225] Rider S.A., Mullins L.J., Verdon R.F., MacRae C.A., Mullins J.J. (2015). Renin Expression in Developing Zebrafish Is Associated with Angiogenesis and Requires the Notch Pathway and Endothelium. Am. J. Physiol.-Ren. Physiol..

[226] Takahashi N., Lopez M.L.S.S., Cowhig J.E.J., Taylor M.A., Hatada T., Riggs E., Lee G., Gomez R.A., Kim H.-S., Smithies O. (2005). *Ren1c* Homozygous Null Mice Are Hypotensive and Polyuric, but Heterozygotes Are Indistinguishable from Wild-Type. J. Am. Soc. Nephrol..

[227] Sequeira-Lopez M.L.S., Nagalakshmi V.K., Li M., Sigmund C.D., Gomez R.A. (2015). Vascular versus Tubular Renin: Role in Kidney Development. Am. J. Physiol.-Regul. Integr. Comp. Physiol..

[228] Hoffmann S., Mullins L., Rider S., Brown C., Buckley C.B., Assmus A., Li Z., Sierra Beltran M., Henderson N., del Pozo J. (2022). Comparative Studies of Renin-Null Zebrafish and Mice Provide New Functional Insights. Hypertension.

[229] Schuchardt A., D’Agati V., Larsson-Blomberg L., Costantini F., Pachnis V. (1994). Defects in the Kidney and Enteric Nervous System of Mice Lacking the Tyrosine Kinase Receptor Ret. Nature.

[230] Schuchardt A., D’Agati V., Pachnis V., Costantini F. (1996). Renal Agenesis and Hypodysplasia in *Ret-k*^–^ Mutant Mice Result from Defects in Ureteric Bud Development. Development.

[231] Heanue T.A., Boesmans W., Bell D.M., Kawakami K., Berghe P.V., Pachnis V. (2016). A Novel Zebrafish *Ret* Heterozygous Model of Hirschsprung Disease Identifies a Functional Role for *Mapk10* as a Modifier of Enteric Nervous System Phenotype Severity. PLoS Genet..

[232] Münch J., Engesser M., Schönauer R., Hamm J.A., Hartig C., Hantmann E., Akay G., Pehlivan D., Mitani T., Akdemir Z.C. (2022). Biallelic Pathogenic Variants in Roundabout Guidance Receptor 1 Associate with Syndromic Congenital Anomalies of the Kidney and Urinary Tract. Kidney Int..

[233] Grieshammer U., Ma L., Plump A.S., Wang F., Tessier-Lavigne M., Martin G.R. (2004). SLIT2-Mediated ROBO2 Signaling Restricts Kidney Induction to a Single Site. Dev. Cell.

[234] Fish J.E., Wythe J.D., Xiao T., Bruneau B.G., Stainier D.Y.R., Srivastava D., Woo S. (2011). A Slit/miR-218/Robo Regulatory Loop Is Required during Heart Tube Formation in Zebrafish. Development.

[235] Wang H., Li Q., Liu J., Mendelsohn C., Salant D.J., Lu W. (2011). Noninvasive Assessment of Antenatal Hydronephrosis in Mice Reveals a Critical Role for *Robo2* in Maintaining Anti-Reflux Mechanism. PLoS ONE.

[236] Wyatt C., Ebert A., Reimer M.M., Rasband K., Hardy M., Chien C.-B., Becker T., Becker C.G. (2010). Analysis of the *Astray/Robo2* Zebrafish Mutant Reveals That Degenerating Tracts Do Not Provide Strong Guidance Cues for Regenerating Optic Axons. J. Neurosci..

[237] Miyasaka N., Sato Y., Yeo S.-Y., Hutson L.D., Chien C.-B., Okamoto H., Yoshihara Y. (2005). *Robo2* Is Required for Establishment of a Precise Glomerular Map in the Zebrafish Olfactory System. Development.

[238] Nishinakamura R., Matsumoto Y., Nakao K., Nakamura K., Sato A., Copeland N.G., Gilbert D.J., Jenkins N.A., Scully S., Lacey D.L. (2001). Murine Homolog of *SALL1* Is Essential for Ureteric Bud Invasion in Kidney Development. Development.

[239] Kawakami H., Bailey A., Gavin E., Gearhart M.D., Isabella A.J., Kawakami Y. (2025). Zebrafish *Sall1a* and *Sall4* Contribute to Body Elongation. Dev. Biol..

[240] Bricaud O., Collazo A. (2006). The Transcription Factor *Six1* Inhibits Neuronal and Promotes Hair Cell Fate in the Developing Zebrafish (*Danio rerio*) Inner Ear. J. Neurosci..

[241] O’Brien J.H., Hernandez-Lagunas L., Artinger K.B., Ford H.L. (2014). MicroRNA-30a Regulates Zebrafish Myogenesis through Targeting the Transcription Factor Six1. J. Cell Sci..

[242] Self M., Lagutin O.V., Bowling B., Hendrix J., Cai Y., Dressler G.R., Oliver G. (2006). Six2 Is Required for Suppression of Nephrogenesis and Progenitor Renewal in the Developing Kidney. EMBO J..

[243] Klesert T.R., Cho D.H., Clark J.I., Maylie J., Adelman J., Snider L., Yuen E.C., Soriano P., Tapscott S.J. (2000). Mice Deficient in Six5 Develop Cataracts: Implications for Myotonic Dystrophy. Nat. Genet..

[244] Davison C., Bedó G., Zolessi F.R. (2022). Zebrafish Slit2 and Slit3 Act Together to Regulate Retinal Axon Crossing at the Midline. J. Dev. Biol..

[245] Aamar E., Dawid I.B. (2010). Sox17 and Chordin Are Required for Formation of Kupffer’s Vesicle and Left-Right Asymmetry Determination in Zebrafish. Dev. Dyn..

[246] Airik R., Bussen M., Singh M.K., Petry M., Kispert A. (2006). *Tbx18* Regulates the Development of the Ureteral Mesenchyme. J. Clin. Investig..

[247] Laquatra C., Sanchez-Martin C., Dinarello A., Cannino G., Minervini G., Moroni E., Schiavone M., Tosatto S., Argenton F., Colombo G. (2021). HIF1α-Dependent Induction of the Mitochondrial Chaperone TRAP1 Regulates Bioenergetic Adaptations to Hypoxia. Cell Death Dis..

[248] Hu P., Deng F.-M., Liang F.-X., Hu C.-M., Auerbach A.B., Shapiro E., Wu X.-R., Kachar B., Sun T.-T. (2000). Ablation of Uroplakin III Gene Results in Small Urothelial Plaques, Urothelial Leakage, and Vesicoureteral Reflux. J. Cell Biol..

[249] Gebauer J.M., Karlsen K.R., Neiss W.F., Paulsson M., Wagener R. (2010). Expression of the AMACO (VWA2 Protein) Ortholog in Zebrafish. Gene Expr. Patterns.

[250] Richardson R.J., Gebauer J.M., Zhang J.-L., Kobbe B., Keene D.R., Karlsen K.R., Richetti S., Wohl A.P., Sengle G., Neiss W.F. (2014). AMACO Is a Component of the Basement Membrane-Associated Fraser Complex. J. Investig. Dermatol..

[251] Kamei C.N., Sampson W.G.B., Albertz C., Aries O., Wolf A., Upadhyay R.M., Hughes S.M., Schenk H., Bonnet F., Draper B.B.W. (2026). Reciprocal Inhibition of Wnt Signaling Pathways Pattern the Interconnection of Epithelial Tubules in the Regenerating Zebrafish Kidney. Development.

[252] Ungar A.R., Kelly G.M., Moon R.T. (1995). *Wnt4* Affects Morphogenesis When Misexpressed in the Zebrafish Embryo. Mech. Dev..

[253] Matsui T., Raya Á., Kawakami Y., Callol-Massot C., Capdevila J., Rodríguez-Esteban C., Izpisúa Belmonte J.C. (2005). Noncanonical Wnt Signaling Regulates Midline Convergence of Organ Primordia during Zebrafish Development. Genes Dev..

[254] Connaughton D.M., Dai R., Owen D.J., Marquez J., Mann N., Graham-Paquin A.L., Nakayama M., Coyaud E., Laurent E.M.N., St-Germain J.R. (2020). Mutations of the Transcriptional Corepressor *ZMYM2* Cause Syndromic Urinary Tract Malformations. Am. J. Hum. Genet..

[255] Dai R., Yin Y., Yu M., Zhang Y., Zhang J., Liu T., Fang X., Wu X., Shen Q., Xu H. (2025). Genitourinary Defects, Anxiety and Aggressive-like Behavior and Glucose Metabolism Disorders in *Zmym2* Mutant Mice with Inserted piggyBac Transposon. Front. Cell Dev. Biol..

