# Supplementary material for: Swimming Upstream to Understand Congenital Anomalies of the Kidney and Urinary Tract: Zebrafish Models for Developmental Biology, Disease Mechanisms, and Functional Interpretation of Genetic Variation
Source: Genes (Basel). 2026 Jul 24;17(8):867. doi: 10.3390/genes17080867 (PMC13536465; doi:10.3390/genes17080867)
Supplement: Supplementary file 1 [file genes-17-00867-s001.zip › genes-4391841-supplementary.pdf]

Supplementary Table S1. Comprehensive list of human CAKUT-associated genes and reported renal phenotypes across model systems.

OMIM Gene ID	Gene Name	Inheritance	Cytoband (human)	OMIM Phenotype	MIM Number	Zebrafish Ortholog(s)	Zebrafish Renal Expression	Modeled in Zebrafish	Zebrafish Renal Phenotype	Embryonic Kidney Expression	Adult Kidney Expression (RNA)	Adult RNA Expression Levels (nCPM)	Adult Bladder Expression (RNA)	Pathway/Function	Other Animal Models	References
ACE	Angiotensin I-Converting Enzyme	AR	17q23.3	Renal tubular dysgenesis	267430	*ace*	Yes - Adult	Yes	No renal phenotype reported	Enriched in endothelial cells.	Proximal tubule–enriched (human RNA; protein consistent)	Low	Low: Fibroblasts and vascular endothelial cells. No protein detected.	Renin–angiotensin signaling (RAS)	Mouse (KO; developmental renal phenotype)	156, 157
AGT	Angiotensin	AR	1q42.2	Renal tubular dysgenesis	267430	*agt*	Yes; stage: Adult.	Yes	No renal phenotype reported	Enriched in rEN+ interstitial cells, interstitial progenitors I, and tubular precursor.	Proximal tubule–enriched (human RNA; protein consistent)	Low	Moderate: Vascular smooth muscle cells. No protein detected.	Renin–angiotensin signaling (RAS)	Mouse (KO; renal vascular abnormalities and tubule atrophy)	158, 159
AGTR1	Angiotensin II Receptor, Type 1	AR	3q24	Renal tubular dysgenesis	267430	*agtr1a; agtr1b*	Yes; both; stage: Adult.	Yes	No renal phenotype reported	Enriched in rEN+ interstitial cells, proliferating interstitial cell, and interstitial cell II.	Enriched in pericytes and fibroblasts (human RNA; protein not detected)	Moderate-high	Moderate: Fibroblasts and smooth muscle cells. No protein detected.	Renin–angiotensin signaling (RAS)	Mouse (*Agtr1a* /*Agtr1b* double KO; renal vascular and papillary/calyceal abnormalities)	158, 160
ARHGEF6	Rho Guanine Nucleotide Exchange Factor 6	XL	Xq26.3	-	–	*arhgef6*	Unknown.	N/A	No renal phenotype reported	Enriched in immune cell, REN+ interstitial cells, endothelial cell.	Enriched in immune cells (human RNA, protein consistent)	Moderate-high	Moderate: Broad expression. Urothelial protein low.	Integrin signaling	Mouse and Xenopus (*Arhgef6* deficiency/KD; duplex/dysplastic kidneys in mouse, pronephric hypoplasia in Xenopus)	161
BNC2	Basonuclin 2	AD	9p22.3-22.2	Lower urinary tract obstruction, congenital	618612	*bnc2*	Yes; site: pronephric tubule; stage: 33 hpf and 100 hpf.	Yes	Distal-outlet obstruction (33hpf), increased apoptosis (33hpf), glomerular cysts (54hpf), dilation of pronephric duct (54hpf)	Enriched in interstitial progenitors II, proliferating interstitial cells, interstitial progenitors.	Proximal tubule–enriched (human RNA; protein low)	Very high	High: Fibroblasts, smooth muscle cells, vascular endothelial cells. Urothelial cell protein low.	Transcriptional regulation	Zebrafish (MO/F0 CRISPR; distal pronephric outlet obstruction and cloacal dilatation); mouse embryonic lower urinary-tract expression reported	57, 162
CHRM3	Cholinergic Receptor Muscarinic, 3	AR	1q43	Prune belly syndrome	100100	*chrm3a; chrm3b*	Unknown.	Yes	No renal phenotype reported	Enriched in endothelial cells.	Vascular endothelial-enriched, with enriched expression in renal collecting tubules and distal convoluted tubule (human RNA; protein low)	High	Very high: Smooth muscle cells. Urothelial cell protein low.	GPCR/calcium signaling	Mouse (KO; lower urinary tract dysfunction with distended bladder and mild hydronephrosis)	163
CHRNA3	Cholinergic Receptor, Neuronal Nicotinic, Alpha Polypeptide 3	AR	15q25.1	Bladder dysfunction, autonomic, with impaired pupillary reflex and secondary CAKUT	191800	*chrna3*	Yes; site: distal expression inferred from *in situ* image; stage: 24 hpf.	Yes	No renal phenotype reported	Enriched in proliferating interstitial cells, interstitial cells II, Interstitial cells III.	No adult expression reported.	Very low/negligible	Very low/negligible. No protein data.	Ion channel	Mouse (KO; impaired detrusor contraction with bladder distension, renal calyx distension, and mild hydronephrosis)	164–166
COL4A1	Collagen, Type IV, Alpha-1	AD	13q34	HANAC syndrome, brain small vessel disease with or without ocular anomalies	611773, 175780	*col4a1*	Yes; site: pronephric tubule; stage: 26 hpf–5 dpf.	Yes	No published renal phenotype in zebrafish	Enriched in rEN+ interstitial cells, endothelial cells, interstitial cells II (ubiquitous except immune cells)	Fibroblast-enriched with vascular/endothelial expression (human RNA; protein low) low expression in tubular cells.	High	High: Fibroblasts, smooth muscle cells, vascular smooth muscle cells, endothelial cells. Urothelial protein low.	ECM/basement membrane	Mouse (heterozygous null reported overtly normal; homozygous null embryonic lethal; dominant missense mutants show renal/glomerular phenotypes)	85, 86, 167–171
CRKL	Crk-like Protooncogene Adaptor Protein	AD	22q11.21	-	–	*crkl*	Yes; site: pronephric tubule; stage: 4.5 dpf.	Yes	Glomerular cysts, pericardial edema, reduced tubule length	Enriched in proliferating interstitial cells, nephron progenitors, tubular precursors (ubiquitous)	Broad expression in collecting duct, tubules, vasculature, and immune cells (human RNA; tubule protein not detected)	Moderate	Moderate: Broad expression. Urothelial cell protein high.	RTK signaling; PI3K/Akt	Mouse (*Crkl* conditional KO; duplicated kidneys/ureters, abnormal UB branching, failed medullary/papillary development, hydronephrosis); zebrafish (*crkl* LOF; reduced pronephric tubule length)	76, 78, 172
DSTYK	Dual Serine/Threonine and Tyrosine Protein Kinase	AD	1q32.1	CAKUT 1	610805	*dstyk*	Yes.	Yes	Cloacal malformation, pericardial edema	Enriched in podocyte precursors, nephron prgneitors, and proliferating interstitial cells (ubiquitous except ureteric epithelial and interstitial progenitor II)	Broad expression in collecting duct, tubules, and vasculature/edothelial cells (human RNA; protein high)	Moderate	Moderate: Broad expression. Urothelial cell protein medium.	FGF signaling	Mouse (*Dstyk* KO; low-penetrance hydronephrosis and hydroureter, proximal ureteric dilation, renal papilla/cortex compression); zebrafish (*dstyk* MO; absent patent pronephric duct opening)	59, 169
ETV4	ETS Variant Transcription Factor 4	AR	17q21.31	-	–	*etv4*	Not detected; site: not detected; stage: Broad posterior mesoderm (not clearly detected in pronephros); Somitogenesis (~16–24 hpf; phenotype at ~24 hpf)	Yes	No renal phenotype reported	Enriched in tubular precursors, ureterix epithelial cells, and nephron progenitors.	Broad expression in collecting duct, tubules, and vasculature/edothelial cells (human RNA; protein medium)	Low	Low: Fibroblasts, smooth muscle, vascular smooth muscle, and urothelial cells. Urothelial protein medium.	Transcriptional regulation	Mouse (*Etv4/Etv5* compound mutants; renal hypodysplasia/agenesis, defective UB branching); zebrafish (*etv4/etv5a* KD; pronephric segmentation/proximal tubule fate defects)	173–175
EYA1	EYA Transcriptional Coactivator and Phosphatase 1	AD	8q13.3	Branchiootorenal syndrome 1, with or without cataracts	113650	*eya1*	No; site: not detected in pronephros (reported)	Yes	No renal phenotype reported	Enriched in nephron progenitors, pretubular aggregate, and tubular precursors.	Enriched in distal convoluted tubule cells (human RNA; no protein data)	Low - Moderate	Moderate: fibroblasts, smooth muscle, and lymphatic endothelial cells. No protein data.	Transcriptional regulation; ureteric bud development	Mouse (*Eya1* KO; renal agenesis/failed metanephric induction; *Eya1/Six1* compound heterozygotes show renal hypoplasia/agenesis)	176–180
FAM58A (CCNQ)	Cyclin Q	XL	Xq28	STAR syndrome	300707	*fam58a*	Unknown.	No	No renal phenotype reported	broadly expressed.	Broad expression in collecting duct, tubules, vasculature, and immune cells (human RNA; glomerular protein low, tubule protein medium)	Low	Moderate: Broad expression. Urothelial protein low.	Cell cycle/proliferation	No CAKUT-relevant animal model identified; mouse *Ccnq* KO/IMPC allele exists but renal/urinary phenotype not reported (MGI)	
FAT4	Fat Atypical Cadherin 4	AR	4q28.1	Van Maldergem syndrome 2	615546	*fat4*	Yes; site: pronephric tubule; stage: 4 dpf.	Yes	Slight pronephric cyst formation (paper is confusing bc in the main paper is says fat1, but not fat4 knockdown, results in pronephric cysts. But in their supplementary, the figure shows an increase in cysts, though less than	Enriched in interstitial cells I & III, proliferating interstitial cells (widespread, except immune cells, ureteric epithelial cells, and nephron progenitors)	Enriched in vascular endothelial cells and fibroblasts, with broad duct/tubular expression (human RNA; glomerular protein medium, tubule protein high)	Moderate-high	Moderate: Smooth muscle, vascular smooth muscle, and endothelial cells. Urothelial cell protein medium.	Hippo signaling	Mouse (*Fat4* mutants/KO; dilated/cystic renal tubules with enlarged kidneys, hydronephrosis and doubled ureters/duplex kidneys; abnormal ureteric budding/excess RET signaling)	64, 79, 181
FGF20	Fibroblast Growth Factor 20	AR	8p22	Renal hypodysplasia/aplasia 2	615721	*fgf20a; fgf20b*	Yes; both; stage: Adult.	Yes	No renal phenotype reported	Enriched in ureteric epithelial cells, tubular precursors, REN+ interstitial cells, and nephron progenitors.	No adult expression reported.	Very low/negligible	Very low/negligible. No protein data.	FGF signaling	Mouse (*Fgf20* KO; smaller kidneys/reduced nephron number; *Fgf9/Fgf20* compound mutants show renal agenesis)	182–186
FOXC1	Forkhead Box C1	AD	6p25.3	Axenfels-Rieger syndrome; anterior segment dysgenesis	602482	*foxc1a; foxc1b*	Yes; *foxc1a* : glomerulus; pronephric duct - inferred; stage: 24–48 hpf.	Yes	No, but only investigated podocytes.	Enriched in podocyte precursor cells, nephron progenitors, interstitial progenitors II.	Enriched in podocytes (human RNA; no protein data)	High	High: Smooth muscle and vascular smooth muscle cells. No protein data.	Transcriptional regulation; ureteric bud development	Mouse (*Foxc1* mutants; ectopic anterior ureteric buds/mesonephric tubules, duplex kidney, abnormal ureters, hydroureter); zebrafish (*foxc1a* KD; reduced podocyte formation/pronephric neck defets)	69, 187–193
FOXC2	Forkhead Box C2	AD	16q24.1	Lymphedema-distichiasis syndrome with renal disease and diabetes mellitus	153400	N/A	N/A.	N/A	N/A	Enriched in podocyte precursors.	Enriched in podocytes, pericytes, lymphatic endothelial cells, vascular smooth muscle, and fibroblasts (human RNA; no protein data)	Moderate	Moderate-High: Lymphatic endothelial cells and vascular smooth muscle cells. No protein data.	Transcriptional regulation; ureteric bud development	Mouse (*Foxc1/Foxc2* compound mutants; renal hypoplasia, ectopic ureteric buds, duplex kidneys, bifid/ectopic/blind-ending ureters, hydroureter; *Foxc2* -null reported glomerular abnormalities); Xenopus (*foxc2* KD; defective pronephric glomus/podocyte	188, 189, 194
FOXP1	Forkhead Box P1	AD	3p13	Intellectual developmental disorder with language impairment with or without autistic features	613670	*foxp1a; foxp1b*	Yes; site: *foxp1b* ; pronephric tubule; stage: 20ss–5 dpf.	Yes	No renal phenotype reported	Enriched in nephron progenitors, podocyte precursors, tubular precursors (broadly expressed except interstitial and interstitial progenitors)	Enriched in renal collecting duct intercalated cells, with broad expression in tubules, vasculature/endothelial cells, and immune cells (human RNA; glomerular protein low, tubule protein	High - Very high	Moderate-High: Broad expression. Urothelial cell protein high.	Transcriptional regulation	Mouse (conditional kidney tubule/collecting duct *Foxp1* deficiency; impaired collecting duct intercalated cell differentiation and distal renal tubular acidosis)	195–198
FRAS1	Fraser Extracellular Matrix Complex Subunit 1	AR	4q21.21	Fraser syndrome 1	219000	*fras1*	Yes; site: pronephric tubule; stage: 24 hpf.	Yes	No renal phenotype reported	Enriched in podocyte precursors, ureteric epitheilal cells, and tubular precursors.	Broad collecting duct and tubule expression with enrichment in connecting tubule cells (human RNA; glomerular protein medium, tubule protein medium)	High - Very high	High: vascular smooth muscle cells. Urothelial cell protein medium.	Integrin signaling	Mouse (*Fras1* null/blebbed mutants; renal agenesis, impaired kidney initiation, glomerular integrity defects)	84, 199, 200
FREM1	FRAS1-Related Extracellular Matrix Protein 1	AR	9p22.3	Bifid nose with or without anorectal and renal anomalies; Manitoba oculotrichoanal syndrome	608980	*frem1a; frem1b*	Yes; *frem1b* ; site: pronephric tubule - inferred; stage: 24 hpf.	No	No renal phenotype reported	Enriched in interstitial cells and tubular precursors.	Broad collecting duct and tubule expression with enrichment in collecting duct intercalated cells, connecting tubule cells, and distal convoluted tubule cells (human RNA; no protein data)	High	Moderate: fibroblasts, smooth muscle and vascular smooth muscle cells. No protein data.	Integrin signaling	Mouse (*Frem1* crf11 p.Ile563Phe missense and *Frem1* eyes2 p.Lys826* truncating alleles; low-penetrance renal agenesis, including compound crf11/eyes2 mutants; renal agenesis enhanced by *Slit2* heterozygosity)	199, 201
FREM2	FRAS1-Related Extracellular Matrix Protein 2	AR	13q13.3	Fraser syndrome 2	617666	*frem2a; frem2b*	Yes; *frem2b* : pronephric duct. *frem2a* : not detected; site: pronephric tubule; stage: 24–48 hpf.	Yes	No renal phenotype reported	Enriched in tubular precursors, ureterix epithelial cells, and podocyte precursors.	Broad collecting duct and tubule expression with enrichment in distal convoluted tubule cells (human RNA; no protein data)	High	Moderate: Urothelial cells. No protein data.	Integrin signaling	Mouse (*Frem2* KO; bilateral renal agenesis with neonatal lethality)	84, 202
GATA3	GATA-Binding Protein 3	AD	10p14	Hypoparathyroidism, sensorineural deafness, and renal dysplasia	146255	*gata3*	Yes; site: distal late tubule - pronephric duct; stage: 15ss–24 hpf.	Yes	Distal pronephric duct defects; reduced/abnormal duct morphology; impaired distal segmentation	Enriched in interstitial cells and ureteric epithelial cells.	Enriched in collecting duct principal cells and papillary tip epithelial cells (human RNA; glomerular protein high, collecting duct protein medium, distal tubules low, proximal tubules high)	High	High: urothelial cells. Urothelial cell protein medium.	Transcriptional regulation; nephric duct development	Mouse (nephric duct-specific *Gata3* KO; ectopic ureteric budding, kidney adysplasia, duplex systems, hydroureter/hydronephrosis, ectopic ureters); zebrafish (*gata3* mutant; abnormal distal late pronephric tubule reported)	32, 43, 67
GFRA1	GDNF Family Receptor Alpha-1	AR	10q25.3	Renal hypodysplasia/aplasia 4	619887	*gfra1a; gfra1b*	Yes; site: *gfra1a* : pronephric duct; gfra1b: weak pronephric duct; stage: 18–20 hpf.	Yes	No renal phenotype reported	Enriched in nephron progenitor cells, ureteric epithelial cells, tubular precursors.	Enriched in fibroblasts (human RNA; glomerular protein low, tubule protein medium)	Moderate	High: Fibroblasts. No protein detected.	RET signaling	Mouse (*Gfra1* KO; bilateral renal agenesis or unilateral/bilateral renal dysgenesis, empty thick-walled bladder; ureteric epithelial conditional KO causes renal agenesis)	203–206
GREB1L	GREB1-Like Retinoic Acid Receptor Coactivator	AD	18q11.1-q11.2	Renal hypodysplasia/aplasia 3	617805	*greb1l*	Yes; site: pronephric tubule; nephric duct; stage: 8.5–11.5 hpf.	Yes	Proximal convoluted tubule swelling	Enriched in proliferating interstitial cells, nephron progenitors, interstitial cells III.	Enriched in collecting duct principal cells, papillary tip epithelial cells, and fibroblasts (human RNA; no protein data)	High	Low-Moderate: Vascular smooth muscle and endothelial cells, urothelial cells, and fibroblasts. No protein data.	Developmental protein, pathways unknown	Mouse (*Greb1l* KO; bilateral kidney agenesis and genital tract anomalies; heterozygotes show decreased ureteric bud branching)	53, 131, 207
GRIP1	Glutamate Receptor-Interacting Protein 1	AR	12q14.3	Fraser syndrome 3	617667	*grip1*	Unknown.	Yes	No renal phenotype reported	Enriched in nephron progenitor, interstitial cells II (widely expressed, not in endothelial cells, interstitial III)	Enriched in collecting duct principal and intercalating cells, and connecting tubule cells (human RNA; glomerular protein medium, tubule protein medium)	Very high	High: Urothelial and smooth muscle cells. Urothelial protein medium.	Integrin signaling	Mouse (*Grip1* KO; renal agenesis/absent kidneys from failed metanephric development)	84, 208
HNF1B	HNF1 Homeobox B	AD	17q12	Renal cysts; renal hypodysplasia; MCDK; syndromic CAKUT (RCAD syndrome)	137920	*hnf1ba; hnf1bb*	Yes; site: *hnf1ba* : NPCs (15ss), Pronephric tubule (20ss-50hpf). *hnf1bb* : NPCs (proximal, 15ss). Proximal tubule 20ss-24hpf; stage: 15ss–24 hpf 50 hpf.	Yes	*hnf1ba* : Pronephric cysts (zfin.org)	Enriched in tubular precursors, ureteric epithelial cells.	Broadly enriched in collecting duct and tubular cells, except podocytes (human RNA; Bowman's capsule, collecting duct, distal, and proximal tubule protein high)	High	Low: Urothelial cells. Protein not detected.	Transcriptional regulation; nephron development	Mouse (*Hnf1b/vHnf1* mutants; defective UB branching, early nephrogenesis, epithelial polarity defects); zebrafish (*hnf1b/vhnf1* mutants; kidney cysts and pronephric segmentation defects)	38, 46, 65, 66, 184, 209, 210
HOXA11	Homeobox A11	AR	7p15.2	Radioulnar synostosis with amegakaryocytic thrombocytopenia 1 (AD)	605432	*hoxa11a; hoxa11b*	Yes; site: *hoxa11a* ; pronephric duct (inferred from zfin). *hoxa11b* : not detected (inferred from zfin); stage: 24 hpf.	No	No renal phenotype reported	Enriched in nephron progenitor cells (differentiating), pretubular aggregates, tubular precursors.	Enriched in fibroblasts and pericytes (human RNA; no protein data)	Low	Low-Moderate: Fibroblasts, smooth muscle and vacular smooth muscle cells, and urothelial cells. Protein not detected.	Transcriptional regulation; ureteric bud development	Mouse (*Hoxa11* single KO: no overt kidney phenotype; *Hoxa11/Hoxd11* double mutants: rudimentary/absent kidneys with defective ureteric bud branching; *Hox11* triple mutants: failure of metanephric kidney induction)	211, 212
HPSE2	Heparanase 2	AR	10q24.2	Urofacial syndrome 1	236730	*hpse2*	Unknown.	No	No renal phenotype reported	Enriched in interstitial cells III, REN+ interstitial cells.	Enriched in fibroblasts, vascular smooth muscle, and pericytes (human RNA; glomerular protein medium, tubule protein medium.	Very high	Very high: Smooth muscle cells. Urothelial cell protein medium.	ECM/basement membrane	Mouse (*Hpse2* KO/mutants; distended bladder/megacystis, abnormal voiding; abnormal bladder/outflow-tract innervation)	213, 214
ITGA8	Integrin, Alpha-8	AR	10p13	Renal hypodysplasia/aplasia 1	191830	*itga8*	Unknown.	Yes	No renal phenotype reported	Enriched in rEN+ interstitial cells, NPCs (differentiating), NPCs.	Enriched in fibroblasts, vascular smooth muscle and endothelial cells (human RNA; glomerular protein high)	High	High: Fibroblasts, smooth muscle cells, vascular smooth muscle cells. Protein not detected.	Integrin signaling	Mouse (*Itga8* KO; delayed/failed UB invasion of metanephric mesenchyme with kidney rudiments and frequent renal agenesis)	215, 216
LRIG2	Leucine-Rich Repeats-and Immunoglobulin-Like Domains-Containing Protein 2	AR	1p13.2	Urofacial syndrome 2	615112	*lrig2*	Unknown.	No	No renal phenotype reported	broadly expressed.	Broad expression in collecting duct, tubules, vasculature, and immune cells (human RNA; tubule protein low)	Moderate	Moderate: Broad expression. Urothelial protein low.	Unknown/poorly defined signaling	Mouse (*Lrig2* mutants; dysfunctional urination/functional bladder outlet obstruction and abnormal bladder innervation)	214
MUC1	Mucin1, Cell Surface Associated	AD	1q22	Tubulointerstitial kidney disease, autosomal dominant, 2	174000	*N/A*	N/A.	N/A	N/A	Enriched in tubular precursors.	Broadly expressed in collecting duct and tubules, except for proximal tubules and podocytes (human RNA; collecting duct protein high, distal tubule protein high)	Low - Moderate	Moderate: Urothelial cells. Urothelial protein medium.	Poorly defined, but evidence suggests cell ahdesion	Mouse *Muc1* KO models exist, but no CAKUT-relevant developmental renal/urinary phenotype identified	217, 218
MYOCD	Myocardin	AD	17p12	Megabladder, congenital	618719	*myocd*	Unknown.	No	No renal phenotype reported	Enriched in rEN+ interstitial cells, interstitial progenitors I, interstitial cells I.	Enriched in vascular smooth muscle cells (human RNA; glomerular protein medium, tubule protein medium)	High	High: Smooth muscle and vascular smooth muscle cells. Urothelial cell protein medium.	Transcriptional regulation	Mouse (*Myocd* loss-of-function compound heterozygotes; congenital megabladder with gross bladder dilation and little/no bladder wall smooth muscle)	219
NPNT	Nephronectin	AR	4q24	-	–	*npnta; npntb*	Yes; site: *npnta* : Pronephric tubule; stage: 20–24 hpf.	Yes	Glomerular filtration defect with proteinuria, podocyte effacement, and GBM structural abnormalities	Enriched in podocyte precursors, ureteric epithelial cells, endothelial cells.	Enriched in podocytes (human RNA; glomerular protein medium)	Very high	Moderate: Vascular endothelial cells. Protein not detected.	Integrin signaling	Mouse (*Npnt* KO/frameshift KI; renal agenesis/hypoplasia, impaired UB invasion/Gdnf signaling); zebrafish (*npnt* KD; podocyte/GBM defects with edema/proteinuria)	82, 83, 87, 147
NRIP1	Nuclear Receptor-Interacting Protein 1	AD	21q11.2-q21.1	Congenital anomalies of kidney and urinary tract 3	618270	*nrip1a; nrip1b*	Yes; site: *nrip1a* : pronehric tubule (zfin inferred); nrip1b: not detected (zfin inferred); stage: 24–48 hpf.	No	No renal phenotype reported	Enriched in endothelial cells, podocyte precursors, immune cells (broadly expressed)	Broadly expressed in collecting duct, tubules, and vasculature/endothelial cells, but enriched in podocytes (human RNA; glomerular protein medium, tubule protein medium)	High	High: Broad expression. Urothelial cell protein medium.	Transcriptional regulation	Mouse (*Nrip1* +/− null heterozygotes; dysplastic kidneys with cystic dilations; one embryo also showed severe hydroureter, hydronephrosis and ureterocele); Xenopus (*nrip1* MO; inhibited pronephros development/distorted pronephric structures with	220

PAX2	Paired Box Gene 2	AD	10q24.31	Papillorenal syndrome	120330	*pax2a; pax2b*	Yes; site: intermediate mesoderm; pronephric tubule; stage: *pax2a* : 1–14ss; 24–48 hpf; *pax2b* : 24 hpf.	Yes	*pax2a* : Complete loss of pronephric tubules due to failed tubule differentiation and disrupted nephron patterning, with preserved glomerulus	Enriched in nPCs (differentiated), pretubular aggregate, tubular precursors.	Broadly expressed in collecting duct and tubules, except for podocytes (human RNA; proximal tubule protein low)	High	Undetected.	Transcriptional regulation; nephric duct development; ureteric bud development	Mouse (*Pax2* ^{1}Neu+/−; renal hypoplasia with increased fetal kidney apoptosis and reduced ureteric bud branching); zebrafish (*pax2a* /no isthmus mutants; defective pronephric tubule/duct epithelial differentiation and cloaca morphogenesis)	36, 38, 221, 222
PBX1	Pre-B-Cell Leukemia Transcription Factor 1	AD	1q23.3	Congenital anomalies of kidney and urinary tract syndrome with or without hearing loss, abnormal ears, or developmental delay	617641	*pbx1a; pbx1b*	Unknown.	Yes	No renal phenotype reported	broadly expressed (except immune cells)	Enriched in vascular endothelial and smooth muscle cells, pericytes, and fibroblasts. Broadly expressed in collecting duct and tubules (human RNA; glomerular protein medium, tubule protein low)	Very high	High: Fibroblasts, smooth muscle cells, vascular endothelial cells, urothelial cells. Urothelial cell protein high.	Transcriptional regulation	Mouse (*Pbx1* KO; renal hypoplasia/mispositioning, unilateral renal agenesis, reduced nephrogenesis/UB branching/ureter elongation)	223, 224
REN	Renin	AR	1q32.1	Renal tubular dysgenesis	267430	*ren*	Yes; site: juxtaglomerular vascular mural cells; stage: 1–5 dpf.	Yes	No renal phenotype reported	Enriched in rEN+ interstitial cells.	Enriched in vascular smooth muscle cells (human RNA; no protein detected)	High	Moderate: Urothelial cells. Protein not detected.	Renin–angiotensin signaling (RAS)	Mouse (*Ren1c* constitutive KO; hydronephrosis, thickened renal arterial walls, renal fibrosis, polyuria/dehydration; heterozygotes normal; conditional Ren models support roles for vascular/tubular renin in kidney development)	225–228
RET	RET Protooncogene	AD	10q11.21	Medullary thyroid carcinoma; Multiple endocrine neoplasia IIA; Multiple endocrine neoplasia IIB; Pheochromocytoma; Hirschsprung	155240, 171400, 162300, 171300, 142623	*ret*	Yes; site: distal late tubule & pronephric duct; stage: 24–48 hpf.	Yes	No clear renal developmental phenotype reported in zebrafish	Enriched in ureteric epithelial cells.	Enriched in macrophages (human RNA; glomerular protein low)	Low	Low: Lymphatic endothelial cells. No protein data.	RET signaling	Mouse (*Ret* KO/*ret* -k– mutants; renal agenesis/hypodysplasia due to defective ureteric bud development)	32, 229–231
ROBO1	Roundabout Guidance Receptor 1	AD	3p12.3	-	–	*robo1*	Unknown.	Yes	No renal phenotype reported	Enriched in nPCs (differentiated), proliferating interstitial cells, endothelial cells (broadly expressed, except immune cells)	Enriched in podocytes and fribroblasts (human RNA; glomerular protein medium, tubule protein medium)	High	High: Fibroblasts, urothelial, smooth muscle, and vascular smooth muscle, and vascular endothelial cells. Urothelial cell protein high.	Guidance signaling	Mouse (*Robo1* p.Ile270Thr ENU model; syndromic CAKUT with hydroureter, duplex/multiplex cystic dysplastic kidneys, hydronephrosis and cystic tubules/collecting ducts)	232–234
ROBO2	Roundabout Guidance Receptor 2	AD	3p12.3	Vesicoureteral reflux 2	610878	*robo2*	Unknown.	Yes	No renal phenotype reported	Enriched in podocyte precursors, NPCs, interstitial cells (broadly expressed except immune cells)	Enriched in podocytes and vascular smooth muscle cells (human RNA; tubule protein high, glomerular protein not detected)	High	High: Fibroblasts. Urothelial protein medium.	Guidance signaling	Mouse (*Robo2* mutants; supernumerary ureteric buds/ectopic kidney induction, antenatal hydronephrosis, high-grade VUR and abnormal UVJ/ureteral orifice development)	233, 235–237
SALL1	SAL-Like 1	AD	16q12.1	Townes-Brocks branchiootorenal-like syndrome; Townes-Brocks syndrome 1	107480	*sall1a; sall1b*	*sall1a* : Yes; *sall1b* unknown; site: *sall1a* : Corpuscle of Stannius; stage: 24–48 hpf.	Yes	No renal phenotype reported	Enriched in nPCs (differentiated), tubular precursors, pretubular aggregates, NPCs.	Enriched in proximal tubule cells and loop of henle epithelial cells (human RNA; glomerular protein low, tubule protein high)	Moderate	Low: fibroblasts. Urothelial protein high.	Transcriptional regulation; ureteric bud development	Mouse (*Sall1* KO; renal agenesis/severe dysgenesis from defective ureteric bud invasion)	32, 238, 239
SIX1	SIX Homeobox 1	AD	14q23.1	Branchiootic syndrome 3	608389	*six1a; six1b*	Not detected (zfin inferred)	Yes	No renal phenotype reported	Enriched in nPCs (differentiated), NPCs, pretubular aggregates.	Enriched in papillary tip epithelial cells (human RNA; protein not detected)	Low	Low: Fibroblasts, urothelial cells. Urothelial cell protein low.	Transcriptional regulation; ureteric bud development	Mouse (*Six1* KO; renal agenesis from failed metanephric mesenchyme/UB outgrowth; *Six1/Eya1* compound heterozygotes show renal hypoplasia/agenesis and blind-ending ureters)	179, 240, 241
SIX2	SIX Homeobox 2	AD	2p21	-	–	*six2a; six2b*	*six2a* : no; *six2b* ; yes; site: intermediate mesoderm and pronephric tubules; stage: 12–18ss.	Yes	Proximal tubule morphogenesis defect (straight tubule) with glomerular abnormalities.	Enriched in nPCs (differentiated), NPCs, pretubular aggregates.	Low podocyte expression (human RNA, no protein data)	Low	Very low: Fibroblasts, vascular smooth muscle and urothelial cells. No protein data.	Transcriptional regulation; ureteric bud development	Mouse (*Six2* KO; severe renal hypoplasia from premature nephron differentiation and depletion of nephron progenitors); zebrafish (*six2b* KD/mutants/F0 CRISPR; proximal pronephros/proximal tubule morphogenesis defects)	62, 68, 242
SIX5	SIX Homeobox 5	AD	19q13.32	Branchiootorenal syndrome 2	610896	*six5*	Unknown.	No	No renal phenotype reported	Enriched in nPCs (differentiated), podocyte precursors, proliferating interstitial cells (broadly expressed except immune cells)	Broadly expressed in collecting duct, tubules, and vasculature/endothelial cells (human RNA; glomerular protein not deteced, tubule protein medium)	Low	Low-Moderate: Fibroblasts, smooth muscle, endothelial, and urothelial cells. Urothelial cell protein medium.	Transcriptional regulation	No CAKUT-relevant animal model identified; *Six5* KO mice reported cataracts, not renal/urinary malformations	243
SLIT2	SLIT Guidance Ligand 2	AD	4p15.31	-	–	*slit2*	Unknown.	Yes	No renal phenotype reported	Enriched in interstitial progenitors I, ureteric epithelial cells, tubular precursors.	Enriched in renal collecting duct intercalated cells (human RNA, no protein detected)	High-Very high	High: Smooth muscle cells and fibroblasts. Protein not detected.	Guidance signaling	Mouse (*Slit2* mutants; supernumerary ureteric buds/multiple ureteric bud outgrowths from the nephric duct)	233, 234, 244
SOX17	SRY-Box 17	AD	8q11.23	Vesicoureteral reflux 3	613674	*sox17*	Unknown.	Yes	No renal phenotype reported	Enriched in endothelial cells.	Enriched in vascular endothelial cells (human RNA; glomerular protein not detected, tubule protein low)	Moderate	High: Vascular endothelial cells. Urothelial cell protein low.	Transcriptional regulation	No CAKUT-relevant animal model identified	245
SRGAP1	SLIT-TOBO Rho GTPase-Activating Protein 1	AD	12q14.2	{Thyroid cancer, nonmedullary, 2}	188470	*srgap1a; srgap1b*	Unknown.	No	No renal phenotype reported	Enriched in podocyte precursors, NPCs (differentiated), tubular precursors (broadly expressed)	Enriched in podocytes and macrophages, with broad expression in collecting duct, tubules, vasculature/endothelial cells (human RNA; no protein data)	High-Very high	High: Fibroblasts. No protein data.	Guidance signaling	No CAKUT-relevant animal model identified	233
TBX18	T-Box Transcription Factor 18	AD	6q14.3	Congenital anomalies of kidney and urinary tract 2	143400	*tbx18*	Not detected (zfin inferred)	No	No renal phenotype reported	Enriched in interstitial progenitors I & II, Interstitial cells II & III, proliferating interstitial cells.	Enriched in fibroblasts, pericytes, and vascular smooth muscle cells (human RNA; no protein data)	Moderate	Moderate-High: Smooth muscle vascular smooth muscle, and fibroblasts. No protein data.	Transcriptional regulation	Mouse (*Tbx18* KO; short hydroureter, hydronephrosis, failed ureteral smooth muscle differentiation)	246
TNXB	Tenascin XB	AD	6p21.33-p21.32	Vesicoureteral reflux 8	615963	*tnxba; tnxbb*	Unknown.	No	No renal phenotype reported	Enriched in interstitial progenitors I, endothelial cells.	Enriched in vascular and lymphatic endothelial cells (human RNA; no protein data)	High	Low: Lymphatic endothelial cells and fibroblasts. No protein data.	Integrin signaling	No CAKUT-relevant animal model identified	
TRAP1	Tumor Necrossi Factor Receptor-Associated Protein 1	AR	16p13.3	-	–	*trap1*	Unknown.	Yes	No renal phenotype reported	broadly expressed.	Broadly expressed (human RNA; glomerular protein medium, tubule protein high)	Moderate	Moderate: Broad expression. Urothelial protein high.	Cell stress response	No CAKUT-relevant animal model identified	19, 247
UPK3A	Uroplakin 3A	AD	22q13.31	-	–	*upk3b (previously upk3a)*	Unknown.	No	No renal phenotype reported	Enriched in immune cells, ureteric epithelial cells.	Enriched in papillary tip epithelial cells (human RNA; glomerular protein not deteced, tubule protein low)	Moderate	High: urothelial cells. Urothelial cell protein high.	Cell adhesion/epithelial organization	Mouse (*Upk3a* KO; enlarged ureteral orifices, VUR, hydronephrosis, urothelial barrier defects)	248
VWA2 (AMACO)	Von Willebrand Factor A Domain-Containing Protein 2	AR	10q25.3	-	–	*vwa2*	Yes; site: pronephros (limited data); stage: 4 dpf.	Yes	No renal phenotype reported	no data.	Low, broad expression in collecting duct and tubules (human RNA; no protein data)	Low	Low: urothelial, vasulcar endothelial, and pericytes. No protein data.	ECM/basement membrane	No CAKUT-relevant animal model identified	249, 250
WNT4	Wingless-Type MMTV Integration Site Family, Member 4	AD	1p36.12	Mullerian aplasia and hyperandrogenism	158330	*wnt4a; wnt4b*	Unknown.	Yes	Perturbed tubule development through inhibition of *wnt4* signaling	Enriched in tubular precursors, NPCs (differentiated), pretubular aggregates, ureteric epithelial cells	Enriched in fibroblasts (human RNA; no protein data)	Low - Moderate	Moderate: Fibroblasts. No protein data.	WNT signaling; nephron development	Mouse (*Wnt4* KO; failed metanephric mesenchyme epithelialization/nephron tubulogenesis)	71, 72, 251–253
ZMYM2	Zinc Finger, MYM-Type 2	AD	13q12.11	Neurodevelopmental-craniofacial syndrome with variable renal and cardiac abnormalities	619522	*zmym2*	Unknown.	No	No renal phenotype reported	broadly expressed (except immune cells)	Broad expression (human RNA; glomerular protein low, tubule protein medium)	High	High: urothelial, endothelial, smooth muscle, and fibroblast cells. Urothelial cell protein high.	Transcriptional regulation	Mouse (*Zmym2* heterozygous LOF/piggyBac models; hydroureter/hydronephrosis, duplex/cystic kidneys, renal agenesis, VUR and duplicated ureteric buds; homozygous piggyBac embryonic lethal); Xenopus (*zmym2* MO; reduced proximal pronephros and cloacal protrusion)	254, 255
